# Nonverbal synchrony in virtual reality

**DOI:** 10.1371/journal.pone.0221803

**Published:** 2019-09-16

**Authors:** Yilu Sun, Omar Shaikh, Andrea Stevenson Won

**Affiliations:** 1 Department of Communication, Cornell University, Ithaca, New York, United States of America; 2 College of Computing, Georgia Institute of Technology, Atlanta, Georgia, United States of America; Max Planck Institute for Human Cognitive and Brain Sciences, GERMANY

## Abstract

How might nonverbal synchrony naturally evolve in a social virtual reality environment? And how can avatar embodiment affect how participants coordinate nonverbally with each other? In the following pre-registered between-subjects experiment, we tracked the movements of pairs of users during a collaborative or competitive task in immersive virtual reality. Each conversational partner controlled either a customized avatar body or an abstract cube that responded to their movements. We compared the movements of the actual user pairs between the two conditions, and to an artificial “pseudosynchrony” dataset composed of the movements of randomly combined participant pairs who did not actually interact. We found stronger positive and negative correlations between real pairs compared to pseudosynchronous pairs, providing evidence for naturally occurring nonverbal synchrony between pairs in virtual reality. We discuss this in the context of the relationships between avatar appearance, task success, social closeness and social presence.

## Introduction

Research on interactions in both virtual reality [1] and face-to-face settings [2] supports the vital role of gestures and posture in communication. However, how this nonverbal behavior is communicated in virtual reality depends both on how user movements are tracked, and how they are rendered. In consumer virtual reality systems, the head and hand tracking required to allow participants to interact with digital content in a virtual space also allows users to see their own movements, and others’ movements, represented by avatars in the virtual environment. This process of tracking and rendering can not only capture user behavior; it can also influence users’ experiences of presence and social closeness, as well as the outcome of the tasks they perform in virtual reality.

The study of avatar representation is long-standing [3] and continues to be the subject of research [4], as it raises questions of customization, diversity, and accuracy in user representation. Considerable research has focused on manipulating the degree to which an avatar appears to be real [5] [4]. Research on how a user’s nonverbal behavior can and should be rendered is also extensive; ranging from gaze [6], to proxemics [7]. Decades of research suggest that when nonverbal behavior is made visible to users in VR, they tend to react to it in ways similar to how they would react to comparable behavior in face-to-face interactions [8]. This would imply that findings from tracked behavior in face-to-face environments should carry over to VR. However, many of the cues available in face-to-face interactions are currently absent or transformed in virtual reality, raising the question of how remaining cues may be interpreted, and how they may affect participants’ naturally emergent nonverbal behavior.

Thus, in this experiment we investigate nonverbal behavior that can be expressed by participants in virtual reality without conscious effort or special equipment; that is closely related to collaborative behavior; and that can be measured by modifying existing techniques taken from face-to-face interactions. Specifically, we ask whether a general measure of nonverbal synchrony may evolve naturally during the course of two-person interactions in virtual reality. We further ask whether, if such naturally occurring synchrony is detectable, whether it is correlated with social closeness and task success, as other studies have found [1]. In addition, we examine the effects of two avatar appearances: an abstract cube and a customized humanoid avatar on synchrony. To do so, we designed two versions of avatars that would provide identical information about participants’ movements. However, only one avatar version appeared humanoid, and the other was a simple cube that changed size and position in response to user movements.

### Nonverbal behavior in virtual environments

Nonverbal behavior in virtual reality not only can reveal things about an individual user’s emotional state and/or personality, but is also linked to the qualities and outcomes of social interaction.

Individuals’ bodily movements reflect their mental processes, allowing observers to perceive aspects of their states of mind, especially emotions [9] [10]. For example, female students who demonstrated side to side (yaw) head movement in a virtual classroom also reported more social anxiety [11]. Emotions have also been identified from dance movement [12] and even from point-light displays which depict an abstracted rendering of an individual only through rendering dots on their major joints [13].

The information derived from body movements is not only useful to conversational partners, it can also influence the outcomes of social interactions. For example, computer algorithm driven agent-avatars that mimicked participants’ head movements were considered more persuasive, and received more positive ratings from participants, compared to agents that did not mimic, even though participants were not aware of the mimicry [14]. In a similar study, participants who interacted with virtual agents whose movements were subtly synchronized reported greater social closeness [1]. These nonverbal cues, though subtle, make a difference in interpersonal communication.

### Nonverbal synchrony

The term “interactional synchrony” was first introduced by Condon and Ogston [15], who used film analysis to understand the behavior patterns of normal participants. Since then, coordinated movement in nonverbal communication has been related to rapport [16] [17] and collaboration [18], leading to positive outcomes in social interactions in a number of domains. For example, movement synchrony coordination was linked to positive outcomes of physician-patient interaction in psychotherapy [19]. A sturdy relationship between rapport and movement synchrony was found among teachers and students in the context of teaching and learning [20]. Parent-infant synchrony is critical to the biological foundation and developmental outcomes of infants [21]. Movement coordination arising from interpersonal synergy [22] not only increases liking and affiliation among individuals [23], but also creates a general prosocial effect [24]. Previous literature has also found that the duration of nonverbal synchrony could be linked to personality traits [25].

Methods of detecting and understanding synchrony have evolved over time. The earliest measures of synchrony used human observers’ subjective ratings to characterize “nonverbal synchrony” [26]. However, capturing and measuring synchrony through human coders is both laborious and can introduce coders’ human biases [27]. Therefore, scholars are constantly evolving methods to quantify and automatically detect synchrony [28] [29] [30] [31] [32] [33]. Recent scholars have been using techniques such as framing-difference methods [34], time series [35], cross-lagged regression [29] and motion energy analysis (MEA) [10] [36], which take advantage of digital video technology to provide quick, general measures of nonverbal synchrony.

Such measures often take the sum or the average of two participants’ movements at points in time and compare the changes in these gross body movements [34]. While such measures do not capture very nuanced behaviors such as mimicry of specific gestures, they do allow the automatic processing of many interactions, and create a measure of synchrony that can be linked to general affect. We thus build off of this research to create a measure of general synchrony applicable to virtual reality.

With the advent of commercial virtual reality equipment, the tracking capabilities of new head-mounted displays (HMDs) and hand-held controllers have made participants’ movement data readily accessible to researchers and have paved the way to create new measures of synchrony, useful for both tracking participants’ actual behavior, and for generating behavior for agent-avatars. Recent work by Shaikh, Sun, and Won [37] and Tarr, et.al [1] have explored how nonverbal synchrony could be captured and visually displayed to better understand dyadic social interactions.

In this study, we extended previous work on collaboration in face-to-face interactions by comparing the movement of collaborative and competitive pairs performing a brainstorming task in virtual reality. The task asked participants to listen to 15 environmental principles, then they were asked to either collaborate or compete with their conversational partners to come up with more ideas related to water or energy conservation. Though attention has been paid to nonverbal synchrony in a collaborative context, not much literature focuses on competition and how this circumstance might disrupt–or increase–interpersonal synchrony [38]. For example, Tschacher, Rees and Ramseyer found “moderate to strong effect sizes for synchrony to occur, especially in the competitive and fun task condition” [39].

Our study is novel in that it examines how pairs of participants’ naturalistic synchronous behaviors may evolve while interacting in collaborative or competitive tasks in virtual reality, a situation that adds additional physical and perceptual constraints and cues, such as wearing heavy head-mounted displays, and holding hand controllers.

### Avatar appearance in virtual reality

Another long-standing area of research in virtual reality is that of avatar appearance. After all, in order for the gestures of participants to have communicative value, they must be represented in some way. Interactions in virtual worlds are often based on those that occur in the physical world. For example, many consumer platforms support [40] or even require [41] users’ adoption of a humanoid avatar. With the help of techniques such as inverse kinematics, users’ gestures are represented through their avatar bodies in ways that match selected nonverbal behavior in the real world [42]. These decisions were supported by research suggesting that realistic avatars were preferred for the illusion of presence [4], that customization and/or a high level of realism enhances some experiences [43], and that matching behavioral realism and avatar appearance is desirable [44].

However, human-appearing avatars are optional. Virtual reality gives us the capability to escape the physical body [45] and this flexibility [46] has inspired a number of experiments on novel embodiment. Although early experiments focused on changing the human appearance of humanoid avatars [47], recent work has explored more radical transformations. Such experiments include providing participants with tails [48] very long arms [49] or even extra limbs [50] to test the limits of embodiment. Participants have been embodied as animals to increases involvement with the nature [51]. Abstract representations, such as Emotibox, have examined how participants could express emotion [3] when they were represented by a featureless box as an avatar.

Recent studies have raised further questions about the nature of embodiment [43]; specifically, how much of the body is required for an embodied experience. In one study, scholars found that an invisible body can still create a sense of body ownership [52]. However, more customized avatars enhanced ownership of the body as well as the feeling of presence in the virtual environment [53]. We thus ask how users’ natural behavior, specifically their nonverbal synchrony, will evolve when both participants are represented by either human or non-human avatars. How does the way that people’s gestures are represented by their avatars affect the outcomes of interactions? And how does the style of gesture representation affect the pair’s perceptions of their interactions?

### Study design

Our study was designed to explore whether and how interpersonal synchrony exists in social interactions in virtual reality when participants performed a collaborative task.

Our first hypothesis predicts that there will be synchrony between participant movements in the collaborative condition as compared to a pseudosynchronous test set, such that there will be significant differences between the correlations of the actual pairs’ movements and the movements of “pseudosynchronous” pairs.

Interpersonal synchrony is often considered in the context of rapport, but people’s movements may be also entrained in other interactions. Thus, while we followed the example of earlier research in using a simple collaborative task [54], we also modified this task in order to create a comparable competitive condition. In both tasks, participant pairs brainstorm environmental principles, and their scores are summed for each pair. Thus, our first research question asks if there will be differences in synchrony between the competitive and collaborative conditions.

Following earlier work on synchrony, our hypothesis is that measures of creative ideation will correlate with a pair’s nonverbal synchrony in a collaborative task. So our second research question asks whether the combined score of pairs in a competitive task will also correlate with their nonverbal synchrony during this task.

To compare the effect of avatar appearance on gestures in a dyadic interaction, it is important to retain as much information about the users’ actual gestures in both kinds of avatars: the humanoid and the abstract avatars. For example, both gaze and expansiveness of gesture are measures that are directly rendered in humanoid avatars from participant movements captured from consumer virtual reality systems. This is in contrast to other gestures (for example, elbow movements, or movements of the torso) which are generally not directly tracked in consumer systems and so must be inferred from the three tracked points of head-mounted display and hand controllers. (Though gaze is not directly tracked in our study, the eyes can be presumed to follow roughly where the head is facing.)

Thus, our third research question asks whether there will be differences in synchrony between pairs controlling humanoid avatars and pairs controlling abstract avatars. In this case, the abstract avatars are specifically designed to convey the same movement dynamics as the humanoid avatars, as in Boker and colleagues [55].

Participant pairs were thus asked to complete either a competitive or a collaborative task, while inhabiting either a personalized humanoid avatar or an abstract cube. This resulted in four experimental conditions: Collaborative Avatar, Collaborative Cube, Competitive Avatar, and Competitive Cube. The study was pre-registered through Open Science Framework https://osf.io/d4vxw/?view_only=466fb762ff3244dc9768d1d90e2934cd.

## Methods and materials

### Participants and data cleaning

96 pairs of participants (192 individuals, 60 males) participated in the study. This study was approved by Cornell Institutional Review Board. The protocol ID number is 1609006582. All participants read and signed consent forms before participating in the study. According to our pre-registered criteria, we excluded one pair of participants’ data because the participant reported motion sickness in the post-test survey. Additionally, we removed one pair of participants who stated that they were “close friends or relatives”. Participants whose movement data for the head or hand was completely missing were also excluded from the results.

After exclusions, there were 152 participants (49 males, 102 females and 1 participant who preferred not to reveal their gender). Participants were randomly assigned to mixed or same-gender pairs, with 33 mixed gender pairs, 8 male-male pairs, and 34 female-female pairs, and 1 pair whose gender composition was not revealed. Participants could select more than one race/ethnicity, so the numbers below total more than the numbers of participants. 69 participants reported themselves as Caucasian, 68 as Asian or Pacific Islanders, 12 as African Americans, 7 people described themselves as more than one race/ethnicity, 8 people described themselves as “other” in this study and 3 people chose “I prefer not to answer this question”. Participants either received course credits or cash compensation for the experiment. By experimental condition, there were 21 collaboration avatar pairs, 20 competition avatar pairs, 17 collaboration cube pairs and 18 competition cube pairs.

### Apparatus

Each participant wore an Oculus Rift head-mounted display with a resolution of 2160 x 1200 at 90 Hz. Participants held the associated Oculus Touch hand controllers throughout the experiment. Participants’ movements were tracked by the head-mounted display and hand controllers. The movement data from the head and hand trackers were stored in a separate database on a local server. The experimental environment was created using the game engine Unity 3D. To keep the experiment as consistent as possible, pre-recorded instructions were automatically played to participants. Additionally, participants’ conversations were recorded as they performed the idea generation task, and later transcribed for coding.

### Data collection procedure

A tracking platform was also necessary to save the movement data from participants in virtual environments. Both at the start of the experimental procedure and at the end, the movement tracking program emitted unique timestamp events to the tracking platform, where they were saved to a database.

During the experimental procedure, the Unity clients would send a snapshot of the participants’ XYZ positional data to the tracking platform at 30 times a second. At the end of the experimental procedure, all snapshots between the two events (automatically defined at the start and end of the procedure) were downloaded to a single CSV file.

Data timestamping was done on the movement tracking platform to avoid discrepancies that might arise by using each clients’ individual system clock. However, a delay in the platform receiving data to timestamp (latency) could also introduce error. In this study, the tracking platform was deployed on the local area network, and not the internet, to help minimize this latency. The setup procedure for the tracking platform, along with its architecture and various features, is thoroughly described in [37].

### Experiment flow

Each participant visited the lab twice. First, they made an initial visit to the lab to be photographed. At a second appointment, they completed two experiences in virtual reality; doing exercises in front of a mirror, and performing an idea generation task brainstorming with a partner. At the end of this visit they completed the post-task survey.

Participants were instructed to go to two different lab rooms on different sides of the building so that they did not see each other in person before the experiment. A research assistant in each room read the initial instructions to the participants, showed them the equipment, and assisted them with the head-mounted display and the hand controllers. In all four conditions, participants were then teleported to a virtual space where there was a plain platform with directional lighting on the top. The space was otherwise featureless to avoid any distractions.

#### First visit to be photographed

Participants came to the lab to get frontal and profile photographs taken before the day of the experiment. The photographs were taken in passport style with no glasses and in neutral lighting.

Then participants were randomly assigned to one of the four experimental conditions. If they were assigned to the humanoid avatar conditions, research assistants then created participants’ virtual avatars based on their photos, using commercial software. The avatar and virtual environment setup procedure followed [56]. If participants were assigned to the abstract cube conditions, they were represented by the same generic cubes with no personalized features for the avatar appearances. The cube got bigger when participants’ arms were moved further apart into a more expansive posture. However, their experience of being photographed before being placed in the experimental environment was identical.

#### Mirror scenario

**Fig 1:**
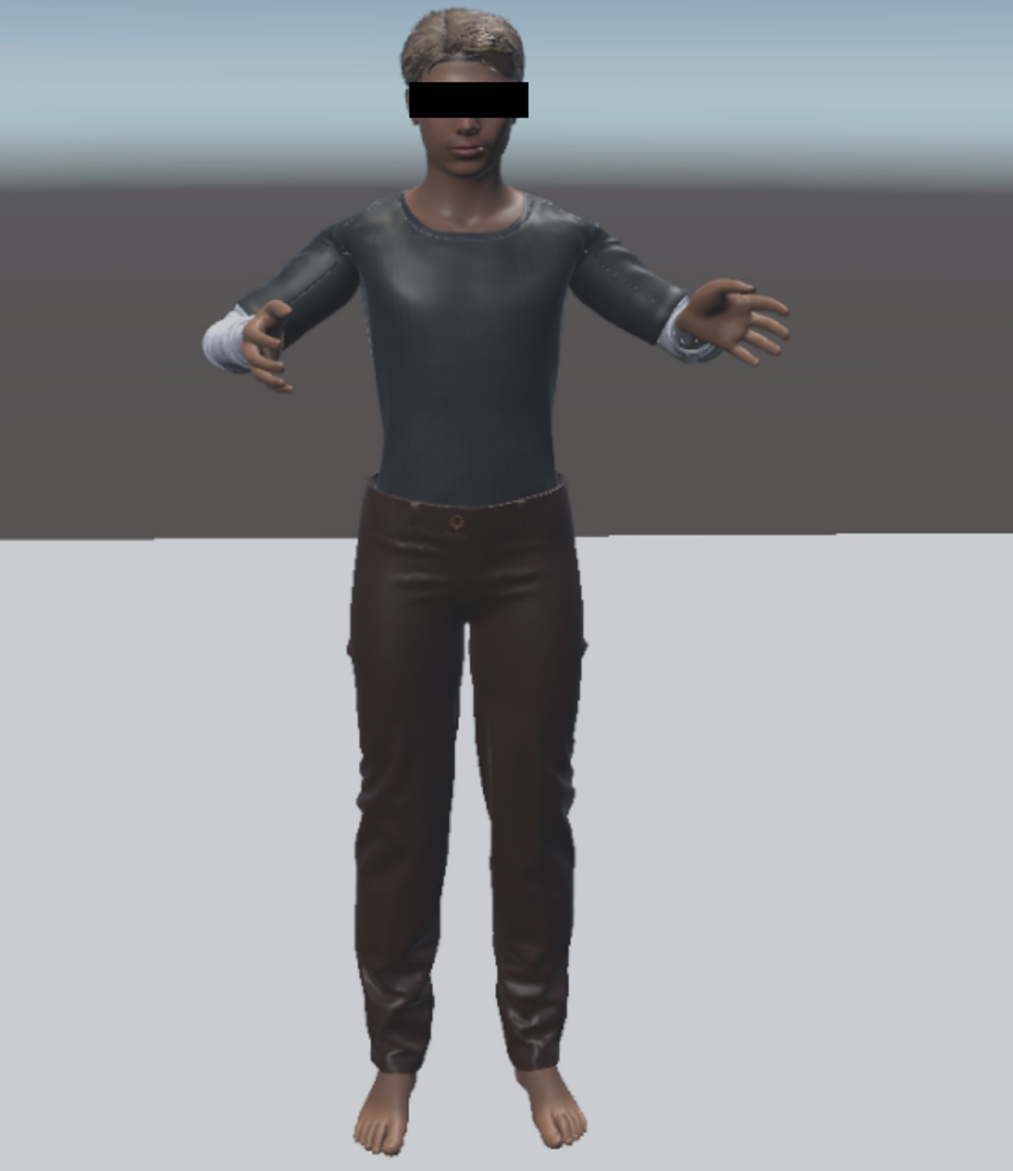
First person view of the humanoid avatar condition. The figure shows the participants views of the mirror scenario. Fig 1 shows a customized humanoid avatar from the first person perspective.

**Fig 2:**
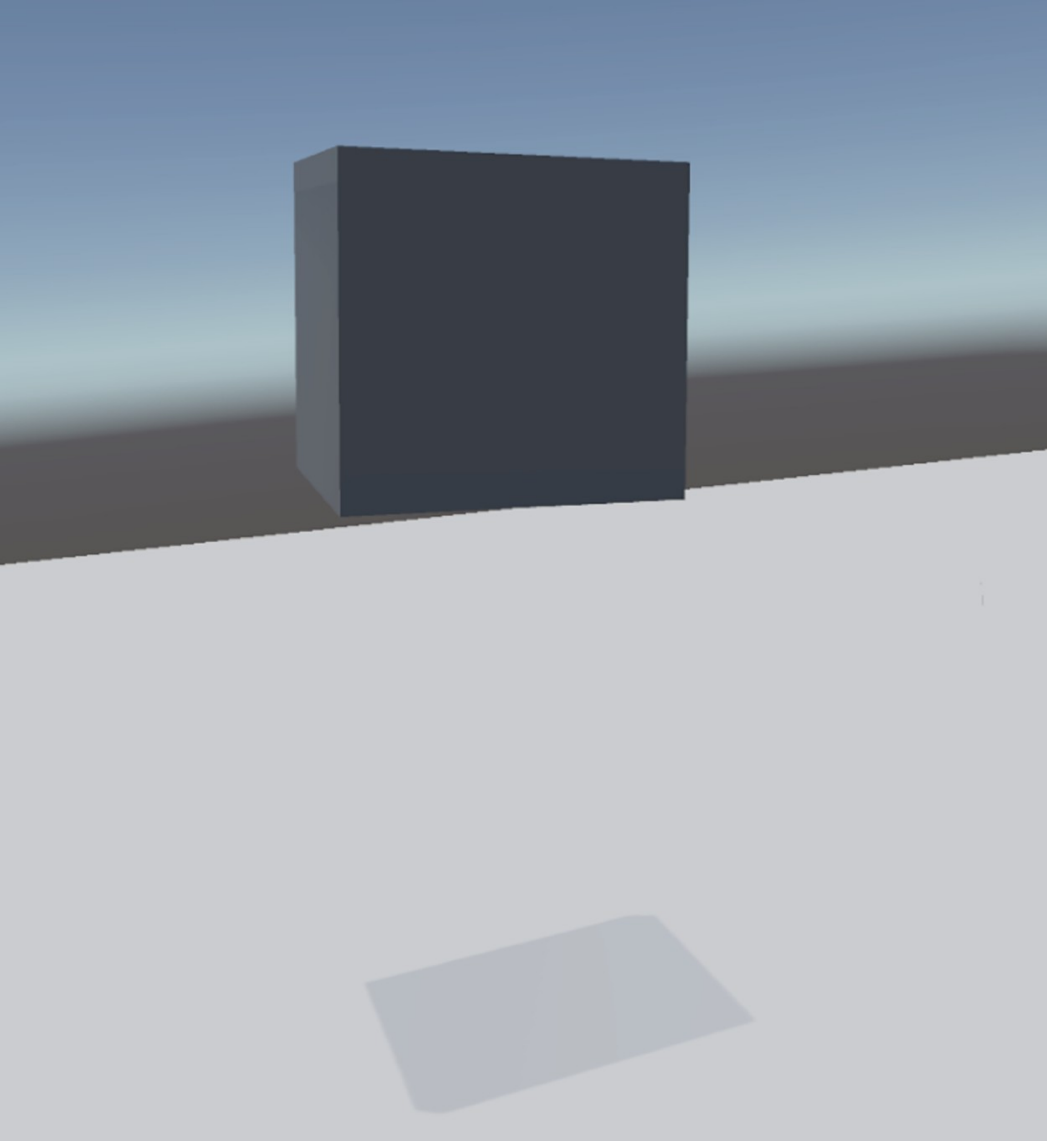
First person view of the abstract cube condition. The figure shows the participants views of the mirror scenario. Fig 2 shows an abstract cube from the first person perspective.

In the mirror scenario, there was a mirror in front of participants in the virtual environment. The participants were instructed to perform three exercises following a metronome called out by a research assistant: raising up their arms, expanding and folding their arms, and stepping toward and away from the mirror. These exercises helped the participants to gain a sense of embodiment in the virtual environment. In the humanoid avatar condition, the exercise showed participants that as they had control over their head and upper body, their avatar hands followed their real hands’ movements. In the abstract cube condition, the exercise showed participants how the volume of the cube shrank or grew when they folded their arms or held them far apart. These exercises also allowed participants to move around the virtual space and observe their avatars following their positional changes. The humanoid avatar and the abstract cube conditions can be seen in Figs 1 and 2.

#### Idea generation task scenario

After checking out their avatar appearances in the mirror scenario, participants were connected to their conversational partners in a networked virtual environment similar to the first scenario, but without the mirrors.

In all conditions, participants then completed an idea generation task with their conversational partners in the virtual environment. They were provided a list of 15 environmental principles from audio instructions related to water use as a prompt. Then they were given 5 minutes to brainstorm more ideas that were not mentioned in the list. These ideas could be related to water use or energy use. When they thought about ideas, they said them out loud. Participants’ conversations were recorded for later transcription and analysis.

**Fig 3:**
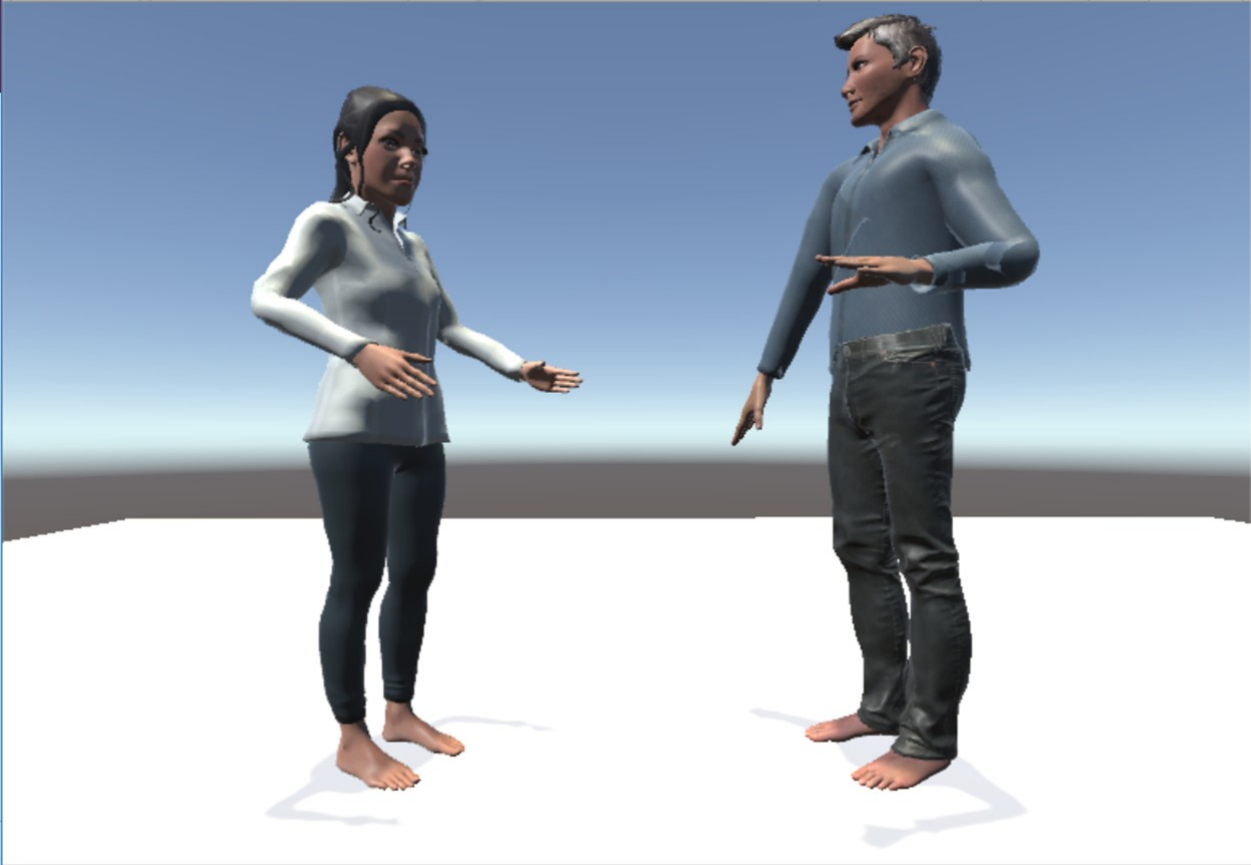
Third person view of the humanoid avatar pairs in the idea generation task scenario. The figure shows two customized humanoid avatars from the third person perspective.

**Fig 4:**
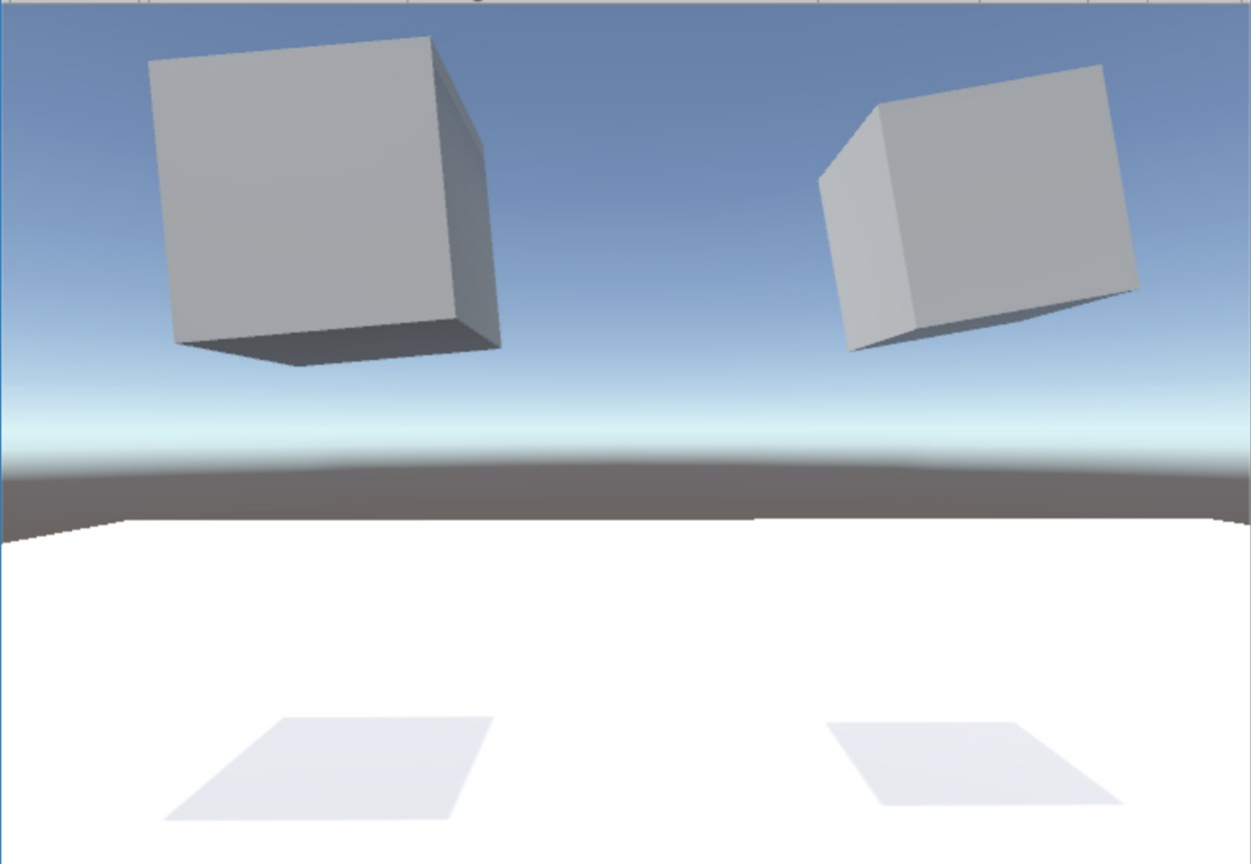
Third person view of the abstract cube pairs in the idea generation task scenario. The figure shows two abstract cubes from the third person perspective.

In the humanoid avatar condition, participants could still see their hands and bodies from the first person perspective. In the abstract cube condition, the participants could see their own shadows as well as their conversational partners who were also represented by cubes. Then they performed the idea generation task for five minutes. When the five minutes timer was up, a chime notified the participants that the idea generation task was finished. After taking off the headset, participants were directed to another laptop to complete a post-test survey. The third person view of the humanoid avatar pairs and abstract cube pairs in the idea generation task scenario can be seen in Figs 3 and 4.

#### Conditions

Participants were randomly placed in one of the two interaction types (collaboration vs. competition) and were represented in one of the two kinds of appearances (humanoid avatar vs. abstract cube). The type of appearance was always the same for both partners. In other words, both participants in a pair either controlled cubes or humanoid avatars. In the cube condition, participants were represented by a white cube with some rendering of nonverbal behavior implemented. For example, the angle of the cube reflected the rotation of participants’ heads. In addition, the volume of the cube would change based on the distance between participants’ hands. When participants stretched their arms to two sides of their bodies respectively, the volume of the cube increased. If participants folded their arms, the volume of the cube decreased. The directional lighting on top of the platform cast a shadow of the cube on the ground so that participants could see the changes in the sizes of the cubes as well as the positional change.

In the humanoid avatar condition, the avatars were customized to resemble the participants, so that when they were in the mirror scenario, they could examine their own appearances. However, in the idea generation task scenario, after the mirror was turned off, participants were represented by generic avatars for scalability of the experiment as creating and rigging customized avatars in the networked environment was time consuming. Participants could not see their faces in this scene, only their hands. Therefore, we matched the light, medium and dark skin tones for the generic avatars, selecting the ones that were closest to participants’ natural skin tone.

Collaborative and competitive conditions differed only in small variations in the verbal instructions to participants. In the collaborative condition, participants were instructed to “generate as many new ideas with their conversational partners together” while in the competition condition, participants were asked to come up with more new ideas “for themselves.” Participants were also told to try to avoid repeating each other’s ideas and, in the competitive condition, the person who came up with the idea first would be credited.

### Measures

#### Synchrony

In order to develop our synchrony measures, we extracted the XYZ positional movement data from each tracker at a rate of 30 times a second. We then summed the distance moved between each time stamp, for each participant, using the measure of Euclidean distance. The data were slightly noisy because the amount of time that the data from each participant took to reach the server varied slightly. In addition, packets of data could also be dropped. This could create error when comparing the distances each participant moved from timestamp to timestamp. In other words, if one of Participant A’s timestamps was dropped, there would be a longer time period between Time 1 and Time 2, and so the distance between Time 1 and Time 2 for Participant A would likely be larger than the distance between Time 1 and Time 2 for Participant B. To address this, we summed the Euclidean distance within every four seconds in order to make equal time comparisons between participants’ movements. Because the total length of participants’ data varied slightly, we removed the final chunks if they were smaller than the time segment used (for example, three seconds when all the other chunks were four seconds). All code for this data processing is available on our pre-registration site.

We then created four measures designed to capture the correlations between each pair of participants’ summed movements. These were the correlations between both participants’ heads, their left hands, their right hands and their total movements (summing up the movement of the left and right hands and head for each participant, for each time period). Because the participants’ Euclidean distance measures for head and hands were not normally distributed, we used Spearman’s Rank Correlation Coefficient to compare the two movement series. These correlations provided us with four “synchrony scores” for each participant pair.

#### Pseudosynchrony

As proposed by Bernieri, Reznick and Rosenthal [33], pseudosynchrony was used to compare the synchrony of real conversational pairs with the synchrony that might happen by chance by randomly pairing up two interactants’ movements from two different conversation pairs. In other words, we correlated the movements of two interactants who never met to create a measure of pseudosynchrony.

We first created pseudosynchronous data sets to compare to the experimental pairs. To do so, we first separated the participant pairs by collaborative and competitive conditions. We then created pseudo-pairs by matching one participant’s data to the data from a participant from the following pair. For example, we would match Participant A from Pair 1 to Participant B from Pair 2. These two participants never interacted, so comparing their movements should produce a correlation purely at random. We repeated this process four times to create four series of correlations for participants’ heads, left hands, right hands and the sums of their total movement.

#### Creativity

The transcribed audio files for each participant were rated independently for novelty and validity by two research assistants based on a grading rubric. Ideas were rated as “valid” if they were reasonable suggestions that did not duplicate the 15 environmental principles as listed in the experiment instructions. If the ideas were not derivative of the fifteen prompts, they were also coded as “novel” and received an additional point. Valid and novel ideas were summed to get the total score for each participant.

Here are some examples of ideas from participants:

- Novel ideas: “using solar energy, windmill”; “carpooling”; “taking public transportation instead of driving”.
- Valid ideas: “collecting and reusing rain water”
- Not valid and not novel ideas: “turn off the tap while brushing the teeth”; “bring a reusable water bottle” These ideas were already mentioned in the fifteen principles.

We used interclass correlation (ICC) to calculate interrater reliability. The reliability for pair validity scores is 0.938 with a 95% confidence interval between 0.903 and 0.961. For pair novel scores reliability was 0.813 with a 95% confidence interval between 0.597 and 0.903. Since there was a high interrater reliability, we took the average of both raters’ scores to create the measure of creativity.

#### Presence

Self-presence and social presence scores were calculated by taking the average of the response from four questions listed in the measure section. First, we checked that the responses were internally consistent among the four questions for self-presence (*alpha* = 0.84) and social presence (*alpha* = 0.82). These presence questions were modified following Gonzalez and Peck [57] and Nowak and Biocca [58] and presented as follows:

Self-presence Questions:

- If something happened to the avatar, it was happening to me.
- The avatar was an extension of me
- The avatar represented my actions well.
- The avatar was me.

Social Presence Questions:

- I felt like the other participant was present.
- I felt like I was in the same room with the other participant.
- I felt like the other participant was aware of my presence.
- I felt like the other participant was real.

Then the self-presence scores and the social presence scores from two participants in a pair were averaged respectively to get the group’s self-presence scores (*M* = 2.277, *SD* = 0.656) and the group’s social presence scores (*M* = 3.086, *SD* = 0.594).

#### Social closeness

Following previous work on social closeness through mediated interactions [59], we asked 10 questions on liking, affiliation, and connectedness (*alpha* = 0.92). We took the average of 10 social closeness questions for each individual and then took the average of both participants’ scores to create a group social closeness measure (*M* = 3.429, *SD* = 0.586).

#### Ranking

Participants were asked to rank three factors in order of the amount of information they provided about their partners’ state of mind. These were: my partner’s choice of words; my partner’s tone of voice; and my partner’s movements as seen in virtual reality.

#### Collaboration/competition manipulation check

As a manipulation check for the collaborative/competitive conditions, participants rated three questions: “How do you feel about working with your partner in the experiment today?”, “How did your conversational partner behave during the experiment?” and “How did you behave during the experiment?” on a scale of 1 to 5 (1 = very collaborative and 5 = very competitive). Participants reported they did not feel more collaborative in the collaborative condition (*M* = 2.145, *SD* = 0.519) than in the competitive condition (*M* = 2.421, *SD* = 0.784), (*W* = 596.5, *p* = 0.179). In answer to the second question, participants in the competitive condition rated their conversational partners’ behavior as more competitive (*M* = 2.395, *SD* = 0.659) compared to ratings in the collaborative condition (*M* = 1.934, *SD* = 0.438), (*W* = 420, *p* < 0.001). In answer to the third question, participants in the competitive condition rated themselves more competitive (*M* = 2.553, *SD* = 0.733) than in the collaborative condition (*M* = 2.171, *SD* = 0.497), (*W* = 506, *p* = 0.021). However, we note that neither condition evoked very strong feelings of competitiveness in participants, as the mean for both conditions remained closer to collaborative than competitive.

#### Appearance manipulation check

As a manipulation check for appearance, participants were also asked “To what extent did you feel that the avatar resembled your partner? Rate on a five point Likert scale from 1 = not at all to 5 = Very strongly”. Participants in the avatar condition (*M* = 2.549, *SD* = 0.568) rated their conversational partners’ avatars as resembling their conversational partners significantly more than in the cube condition (*M* = 1.529, *SD* = 0.675), (*W* = 1233.5, *p* < 0.001).

## Results

### Nonverbal synchrony

We begin by examining our hypotheses regarding naturally occurring synchrony, as follows:

H1: There will be synchrony between participant movements in the collaborative condition as compared to a pseudosynchronous test set, such that correlations between the collaborative pairs’ movements and the pseudosynchronous movements will differ statistically significantly using an independent t-test.

In order to address this question, we compared the actual synchrony scores of the collaborative group to equivalent pseudosynchrony scores. Because the right and left hand data were not normally distributed, we used a Wilcoxon rank sum test to compare the real and pseudosynchronous data sets.

There were significant differences between the collaborative pairs and the pseudosynchronous pairs in terms of the left hand and total movement (see Table 1), such that the head movements of the true collaborative pairs were positively correlated between participants, but hand movements for both the right and left hand were negatively correlated. However, only correlations for the left hand and total movement were statistically significantly different from the pseudosynchronous condition. There was considerably more variance for the competitive pairs, so differences were not statistically significant, as seen in Table 2; however, the direction of the correlations was similar.

**Table 1:** Summary statistics and Wilcoxon rank sum test for four-second summed distance in collaborative condition.

Body Region	Synchrony	Pseudosynchrony	Wilcox.test
Head (*n* = 38)	*M* = −0.006, *SD* = 0.144	*M* = 0.006, *SD* = 0.123	*W* = 676, *p* = 0.638
Left Hand (*n* = 31)	*M* = −0.089, *SD* = 0.174	*M* = 0.024, *SD* = 0.139	*W* = 293, *p* = 0.008
Right Hand (*n* = 33)	*M* = −0.077, *SD* = 0.191	*M* = 0.011, *SD* = 0.126	*W* = 400, *p* = 0.065
Total (*n* = 31)	*M* = −0.084, *SD* = 0.190	*M* = 0.021, *SD* = 0.129	*W* = 321, *p* = 0.024

Table 1 shows the mean and standard deviation of the synchrony and pseudosynchrony scores (derived using Spearman correlations) for the four-second summed distance in the collaborative condition for each body region, as well as a Wilcoxon rank sum test to check whether there is a statistically significant difference between the synchrony and pseudosynchrony pairs. If participants had missed some but not all data for a body region, then we excluded that body region, but kept the other two regions for analyses. For example, if Participant 1 had dropped tracking for the first 30 seconds of the left hand, only the synchrony scores for the right hand and head would be recorded.

**Table 2:** Summary statistics and Wilcoxon rank sum test for four-second summed distance in competitive condition.

Body Region	Synchrony	Pseudosynchrony	Wilcox.test
Head (*n* = 38)	*M* = 0.010, *SD* = 0.192	*M* = 0.049, *SD* = 0.135	*W* = 597.5, *p* = 0.198
Left Hand (*n* = 36)	*M* = −0.126, *SD* = 0.151	*M* = 0.008, *SD* = 0.145	*W* = 364, *p* = 0.001
Right Hand (*n* = 35)	*M* = −0.157, *SD* = 0.217	*M* = 0.009, *SD* = 0.184	*W* = 372, *p* = 0.004
Total (*n* = 34)	*M* = −0.150, *SD* = 0.201	*M* = 0.019, *SD* = 0.170	*W* = 334, *p* = 0.002

Table 2 shows the mean and standard deviation of the synchrony and pseudosynchrony scores (derived using Spearman correlations) for the four-second summed distance in the competitive condition for each body region, as well as a Wilcoxon rank sum test to check whether there is a statistically significant difference between the synchrony and pseudosynchrony pairs. If participants had missed some but not all data for a body region, then we excluded that body region, but kept the other two regions for analyses. For example, if Participant 1 had dropped tracking for the first 30 seconds of the left hand, only the synchrony scores for the right hand and head would be recorded.

Before we pre-registered the study, we used Pearson R correlations between the two participants to develop our measure of synchrony, and this decision is reflected in the code that we uploaded to Open Science Framework. However, when we ran the pre-registered study, we found that the movement data for most participants was not normally distributed. For this reason, we switched to use the Spearman correlation as more appropriate for our main analysis.

We note that the direction and significance of the results remain essentially the same whether Spearman or Pearson are used. However, in order to remain consistent with the pre-registration, we also reproduce Tables 1 and 2 using the Pearson correlations in Appendix A.

#### Lagged synchrony scores

**Fig 5:**
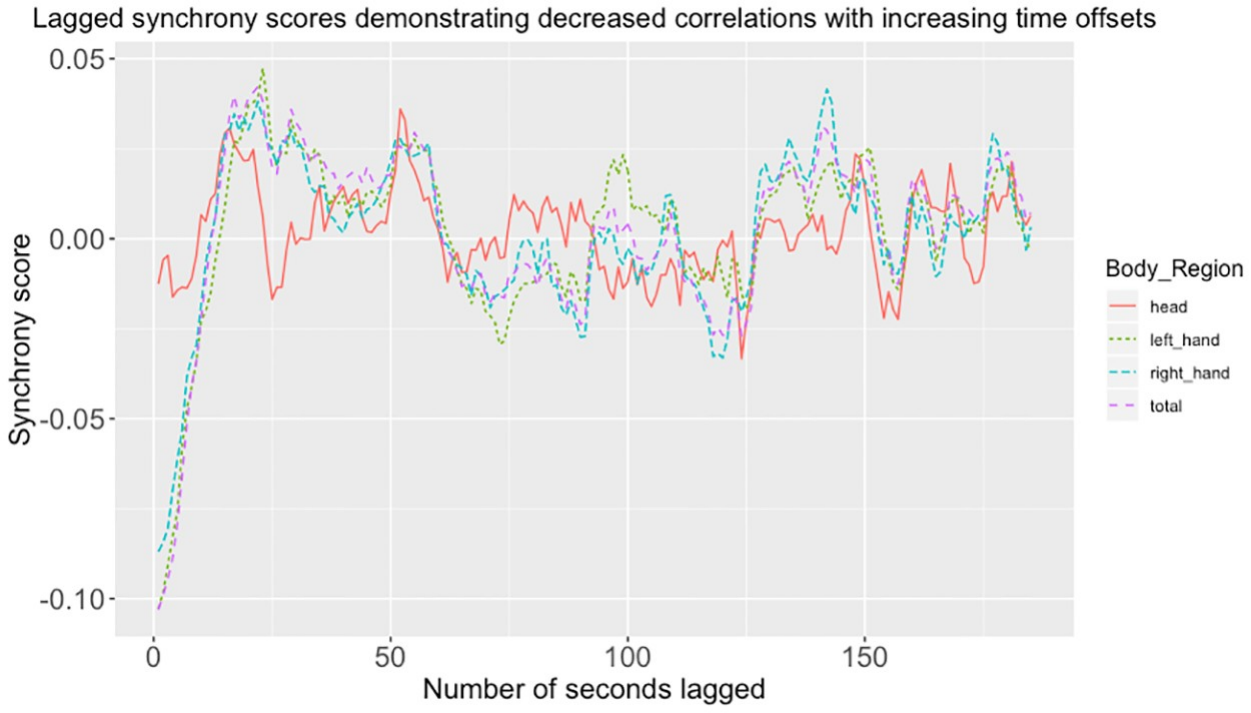
Lagged synchrony scores for different body regions. This figure shows the results of lagging participants’ movements as an alternate method of establishing synchrony. Head movements, hands movements, and total movements, each represented by one line. At time 1 on the X-axis, the Y-axis shows the average of the correlations between the first two 100-second segments of each participant’s movements. These correlations are then offset in two directions. Participant A’s movements were moved forward one second, such that their 100-second segment movement traces starting at time one are matched successively to Participant B’s 100-second segment movements starting at times two, three, etc., up to 185 seconds. This period of 185 seconds was chosen to maximize the number of eligible pairs. Because the length of each recording varied slightly, such that after 185 seconds some participant pairs were not recorded. The process was then reversed, such that Participant B’s movements were moved forward one second while Participant A’s remained stationary. We then averaged all the correlations at each lag; A on B, and B on A. The X-axis shows the amount of offset (averaged across both directions) at increasingly large amounts of time, from 0 seconds to 185 seconds. We see a strong negative Spearman’s: correlation at the beginning, which levels off at around 25 seconds offset.

As an alternate method of demonstrating synchrony, we explored the effects of lagging synchrony scores following Paxton and Dale’s procedure [38]. To do this we created a series of correlations when the two participants’ movements first were lined up perfectly, and then lagged at intervals increasing by one second. Fig 5 illustrates this procedure, with each point representing the average of Participant A lagged on Participant B, and Participant B lagged on Participant A.

#### One to four second synchrony increment

**Fig 6:**
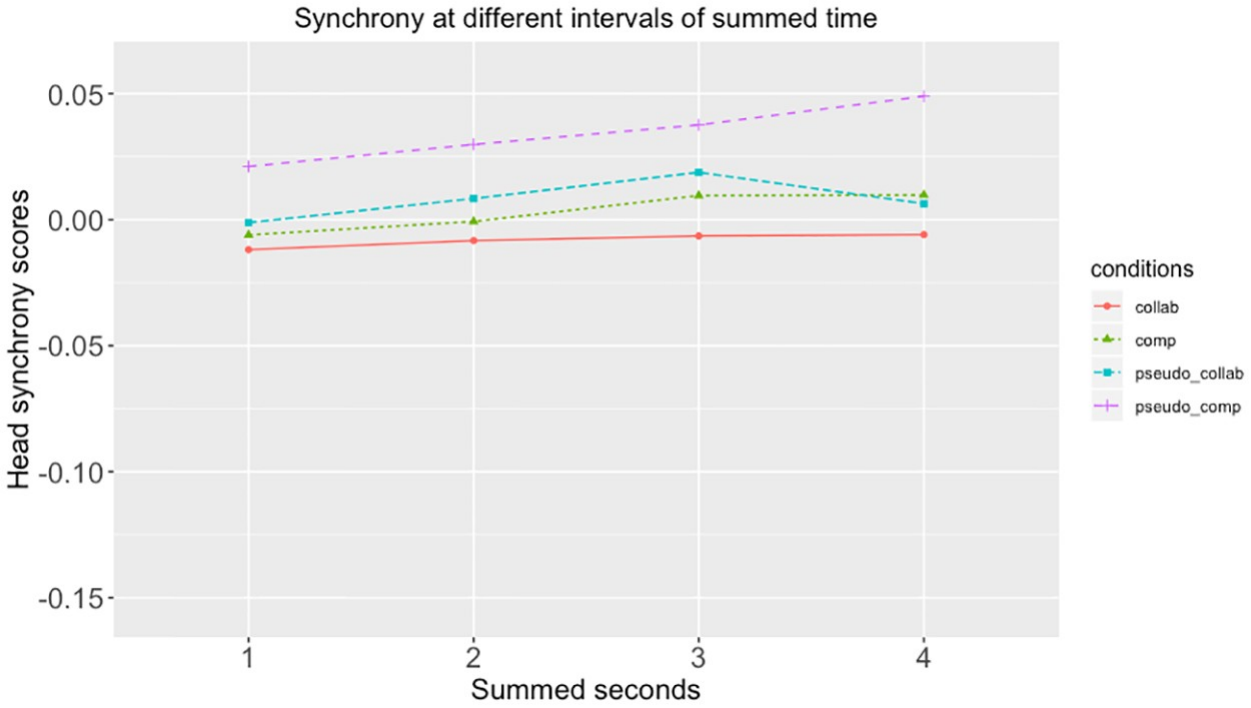
Head synchrony scores at different intervals of summed time. This figure shows the head synchrony scores at when summed with 1 second, 2 seconds, 3 seconds, and 4 seconds.

**Fig 7:**
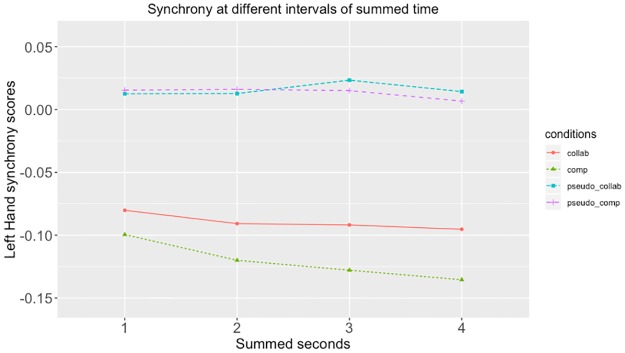
Left hand synchrony scores at different intervals of summed time. This figure shows the left hand synchrony scores at when summed with 1 second, 2 seconds, 3 seconds, and 4 seconds.

**Fig 8:**
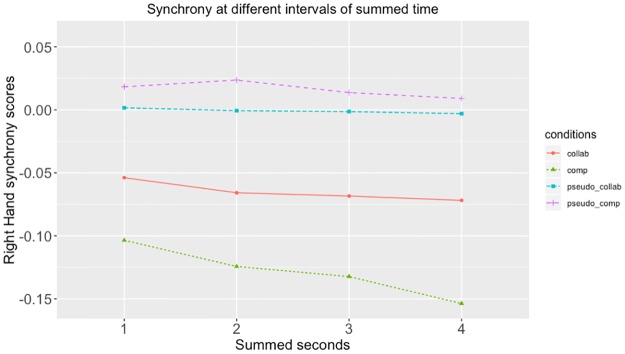
Right hand synchrony scores at different intervals of summed time. This figure shows the right hand synchrony scores at when summed with 1 second, 2 seconds, 3 seconds, and 4 seconds.

**Fig 9:**
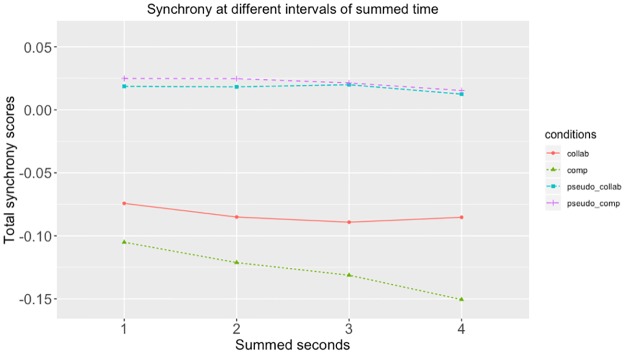
Total synchrony scores at different intervals of summed time. This figure shows the total synchrony scores when summed with 1 second, 2 seconds, 3 seconds, and 4 seconds.

At reviewer suggestion, we added an additional, exploratory analysis, examining the differences between real and pseudosynchronous pairs when movement was summed at different increments. Tables 3 and 4 show the differences between collaborative and competitive conditions when movements were summed at one second increments. These indicate that the patterns shown at 4 second increments are consistent at smaller increments. Figs 6–9 show these data at 1, 2, 3 and 4 second increments for the head, left hand, right hand and total synchrony scores.

**Table 3:** Summary statistics and Wilcoxon rank sum test for one-second summed distance in collaborative condition.

Body Region	Synchrony	Pseudosynchrony	Wilcox.test
Head (*n* = 38)	*M* = −0.012, *SD* = 0.095	*M* = −0.001, *SD* = 0.085	*W* = 684, *p* = 0.699
Left Hand (*n* = 31)	*M* = −0.082, *SD* = 0.123	*M* = 0.023, *SD* = 0.090	*W* = 242, *p* < 0.001
Right Hand (*n* = 33)	*M* = −0.060, *SD* = 0.131	*M* = 0.013, *SD* = 0.094	*W* = 367, *p* = 0.023
Total (*n* = 31)	*M* = −0.074, *SD* = 0.138	*M* = 0.023, *SD* = 0.084	*W* = 282, *p* = 0.005

Table 3 shows the mean and standard deviation of the synchrony and pseudosynchrony scores from a one-second summed distance method in the collaboration condition for each body region, as well as a Wilcoxon rank sum test to check whether there is a statistically significant difference between the synchrony and pseudosynchrony pairs. If participants had missed some but not all data for a body region, then we excluded that body region, but kept the other two regions for analyses. For example, if Participant 1 had dropped tracking for the first 30 seconds of the left hand, only the synchrony scores for the right hand and head would be recorded.

**Table 4:** Summary statistics and Wilcoxon rank sum test for one-second summed distance in competitive condition.

Body Region	Synchrony	Pseudosynchrony	Wilcox.test
Head (*n* = 38)	*M* = −0.006, *SD* = 0.136	*M* = 0.021, *SD* = 0.083	*W* = 633, *p* = 0.36
Left Hand (*n* = 36)	*M* = −0.093, *SD* = 0.118	*M* = 0.017, *SD* = 0.104	*W* = 342, *p* < 0.001
Right Hand (*n* = 35)	*M* = −0.107, *SD* = 0.163	*M* = 0.016, *SD* = 0.125	*W* = 386, *p* = 0.007
Total (*n* = 34)	*M* = −0.108, *SD* = 0.158	*M* = 0.025, *SD* = 0.125	*W* = 330, *p* = 0.002

Table 4 shows the mean and standard deviation of the synchrony and pseudosynchrony scores for the one-second summed distance in the competitive condition for each body region, as well as a Wilcoxon rank sum test to check whether there is a statistically significant difference between synchrony and pseudosynchrony pairs. If participants had missed some but not all data for a body region, then we excluded that body region, but kept the other two regions for analyses. For example, if Participant 1 had dropped tracking for the first 30 seconds of the left hand, only the synchrony scores for the right hand and head would be recorded.

Our first research question asked whether the movements of participants in a collaborative task would be more synchronous than those in a competitive task.

RQ1: In the collaborative setting, will synchronous movement differ compared to the competitive condition, such that correlations between the collaborative pairs’ movements and the pseudo pairs movements will differ statistically significantly using an independent t-test?

We used non-parametric t-tests to compare conditions across the different body regions, but found no significant differences between the collaborative and competitive pairs for synchrony scores by any body region. In fact, the correlations for competitive pairs were slightly stronger (both more positive and more negative) overall.

Given that the largest correlations were negative, we suggest that the movements captured by the tracking data may have represented turn-taking behavior. Examples of data with strong negative and positive correlations for head, hands, and total movement can be seen in Figs 10–17.

**Fig 10:**
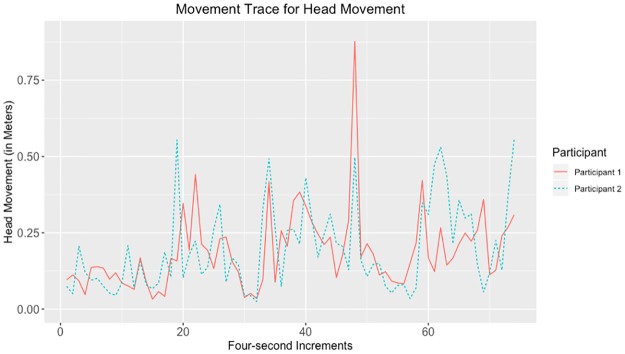
Movement trace for highly positive head synchrony. This figure shows positive head synchrony with the Spearman correlation rho value of 0.494.

**Fig 11:**
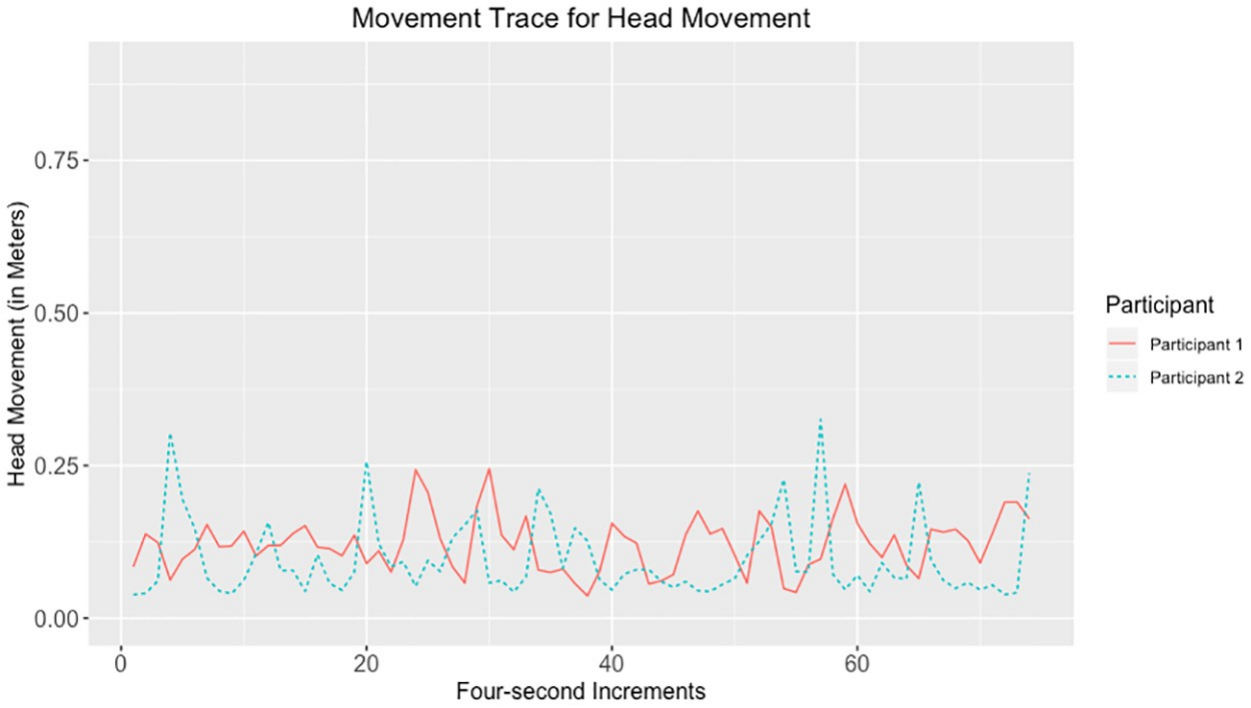
Movement trace for highly negative head synchrony. This figure shows negative head synchrony with the Spearman correlation rho value of −0.374.

**Fig 12:**
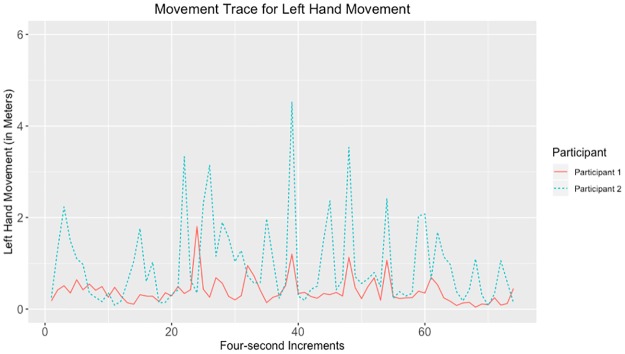
Movement trace for highly positive left hand synchrony. This figure shows positive left hand synchrony with the Spearman correlation rho value of 0.245.

**Fig 13:**
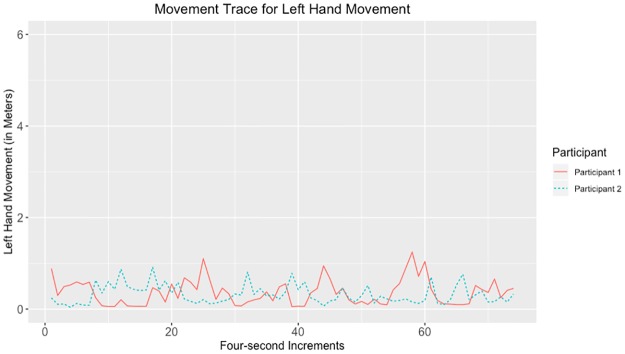
Movement trace for highly negative left hand synchrony. This figure shows negative left hand synchrony with the Spearman correlation rho value of −0.515.

**Fig 14:**
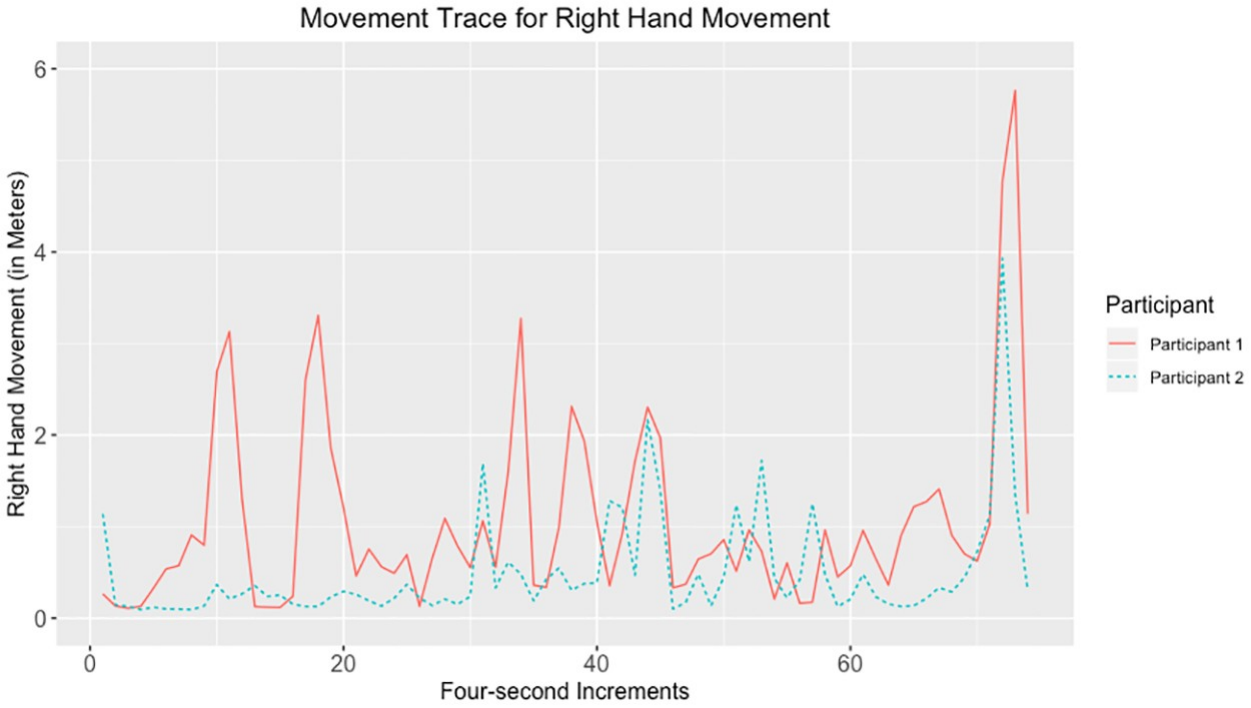
Movement trace for highly positive right hand synchrony. This figure shows positive right hand synchrony with the Spearman correlation rho value of 0.271.

**Fig 15:**
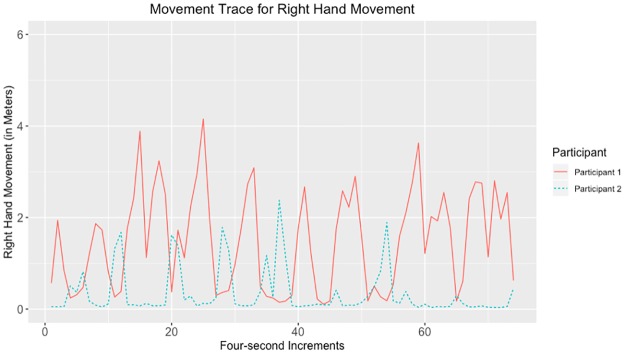
Movement trace for highly negative right hand synchrony. This figure shows negative right hand synchrony with the Spearman correlation rho value of −0.631.

**Fig 16:**
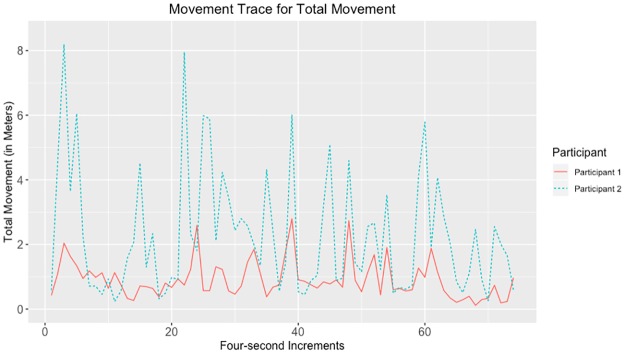
Movement trace for highly positive total synchrony. This figure shows positive total synchrony with the Spearman correlation rho value of 0.289.

**Fig 17:**
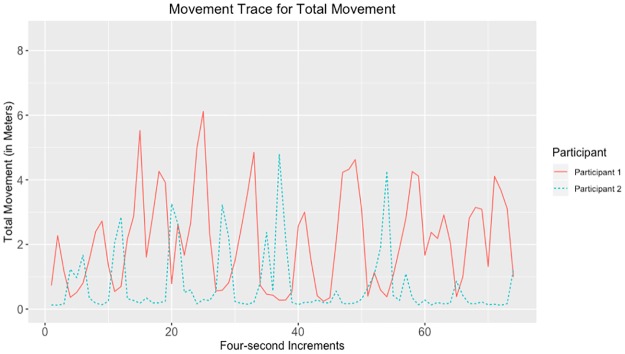
Movement trace for highly negative total synchrony. This figure shows negative total synchrony with the Spearman correlation rho value of −0.577.

### Synchrony and creativity

Our second hypothesis examined the relationship between synchronous movement and creative ideation.

H2: Synchronous movement as tracked during the collaborative task and defined by correlations between the participants’ summed movements will correlate statistically significantly with the total score of both participants in a pair.

We also asked a second question regarding the participants in the competitive task:

RQ2: In the competitive setting, will synchronous movements positively correlate with total score in a creative task?

We cannot reject the null hypothesis for H2, as we did not find significant correlations between the synchrony scores and total scores (*M* = 17.952, *SD* = 7.401) in the collaborative task (all *p’s* > .25, using Spearman’s Ranked Correlation Coefficient).

Regarding RQ2, we also did not find any statistically significant relationships between synchrony movements from the competitive condition (*M* = 20.434, *SD* = 6.539) and the total scores of those pairs (all *p’s* > .15, using Spearman’s Ranked Correlation Coefficient).

### Synchrony and appearance

For our third research question, we investigated the interaction effects of avatar appearance and social closeness or social presence, asking whether participants who were controlling customized humanoid avatars would have measurably more synchrony than participants controlling abstract cube avatars. We limited this question to the cooperative condition where we predicted synchrony would appear.

The summary statistics for the synchrony scores in the Collaborative Avatar and Collaborative Cube conditions were listed in Table 5. Using a Wilcoxon rank sum test, we did not find a significant difference between the two avatar appearance conditions (See Table 5). Therefore, for the remaining exploratory analyses in the next section, we grouped all four conditions together.

**Table 5:** Summary statistics and Wilcoxon rank sum test for collaborative avatar and cube conditions.

Body Region	Collaborative Avatar	Collaborative Cube	Wilcox.test
Head	*n* = 21, *M* = −0.006, *SD* = 0.165	*n* = 17, *M* = −0.006, *SD* = 0.119	*W* = 180, *p* = 0.977
Left Hand	*n* = 17, *M* = −0.071, *SD* = 0.184	*n* = 14, *M* = −0.110, *SD* = 0.165	*W* = 137, *p* = 0.493
Right Hand	*n* = 19, *M* = −0.069, *SD* = 0.202	*n* = 14, *M* = −0.087, *SD* = 0.183	*W* = 133, *p* = 1
Total	*n* = 17, *M* = −0.058, *SD* = 0.199	*n* = 14, *M* = −0.115, *SD* = 0.181	*W* = 140, *p* = 0.421

Table 5 shows the mean and standard deviation of the synchrony scores in the collaborative avatar and collaborative cube conditions for each body region, as well as a Wilcoxon rank sum test to check whether there is a statistically significant difference in the synchrony scores for different avatar appearances.

A linear regression model from R’s lm package was used to test whether the relationship between the variables was significant.

RQ 3a: Will there be an interaction between appearance, liking (measured as “social closeness”) and cooperative condition, such that more or less synchrony is seen in pairs using realistic avatars?

There was no interaction between appearance and social closeness in the collaboration condition that affects total synchrony in participants’ interactions (*β* = 0.618, *p* = 0.567). As an exploratory analysis, we also asked whether there was a similar interaction effect in the competitive condition. However, we did not find a significant relationship between the total synchrony score and social closeness in the competitive condition when the avatar is a cube as opposed to a humanoid avatar (*β* = −1.72, *p* = 0.145).

RQ 3b: Will there be an interaction between appearance, social presence and cooperative condition, such that more or less synchrony is seen in pairs using realistic avatars?

There was no significant interaction effect with synchrony, avatar appearance and social presence scores in the collaborative or competitive conditions.

### Exploratory analyses of social presence and social closeness

#### Social presence and ranking

After the experiment, the participants were asked to drag and drop three factors to rank them in the order that gave them the most information about their partner’s state of mind. Overall, the majority of participants (92 people) rated “tone of voice” as the top factor that gave them information about their conversational partner. Choice of words was rated as second most important (83 people), followed by participants’ movements as seen in VR which was only selected as a first-choice by 4 participants. We note that 26 participants did not change the order of the factors, which was choice of words, tone of voice, and movement as seen in VR, which meant that their answer was not recorded. We thus did not include their answer in this analysis, but it is possible that they did not reorder the factors because they agreed with the default order. Full details on the responses are available in Table 6.

**Table 6:** Ranking.

Condition	Choice of Words	Tone of Voice	Movement in VR
Collaborative Avatar	Rank 1st: 10	Rank 1st: 24	Rank 1st: 1
	Rank 2nd: 23	Rank 2nd: 10	Rank 2nd: 2
	Rank 3rd: 2	Rank 3rd: 1	Rank 3rd: 32
Collaborative Cube	Rank 1st: 5	Rank 1st: 21	Rank 1st: 0
	Rank 2nd: 20	Rank 2nd: 5	Rank 2nd: 1
	Rank 3rd: 1	Rank 3rd: 0	Rank 3rd: 25
Competitive Avatar	Rank 1st: 5	Rank 1st: 27	Rank 1st: 0
	Rank 2nd: 22	Rank 2nd: 4	Rank 2nd: 6
	Rank 3rd: 5	Rank 3rd: 1	Rank 3rd: 26
Competitive Cube	Rank 1st: 10	Rank 1st: 20	Rank 1st: 3
	Rank 2nd: 18	Rank 2nd: 10	Rank 2nd: 5
	Rank 3rd: 5	Rank 3rd: 3	Rank 3rd: 25

Table 6 shows the number of people who ranked “choice of words”, “tone of voice” and “movement” from the most to least important factors that gave them the most information about their conversational partners’ state of mind.

#### Synchrony and social closeness

**Fig 18:**
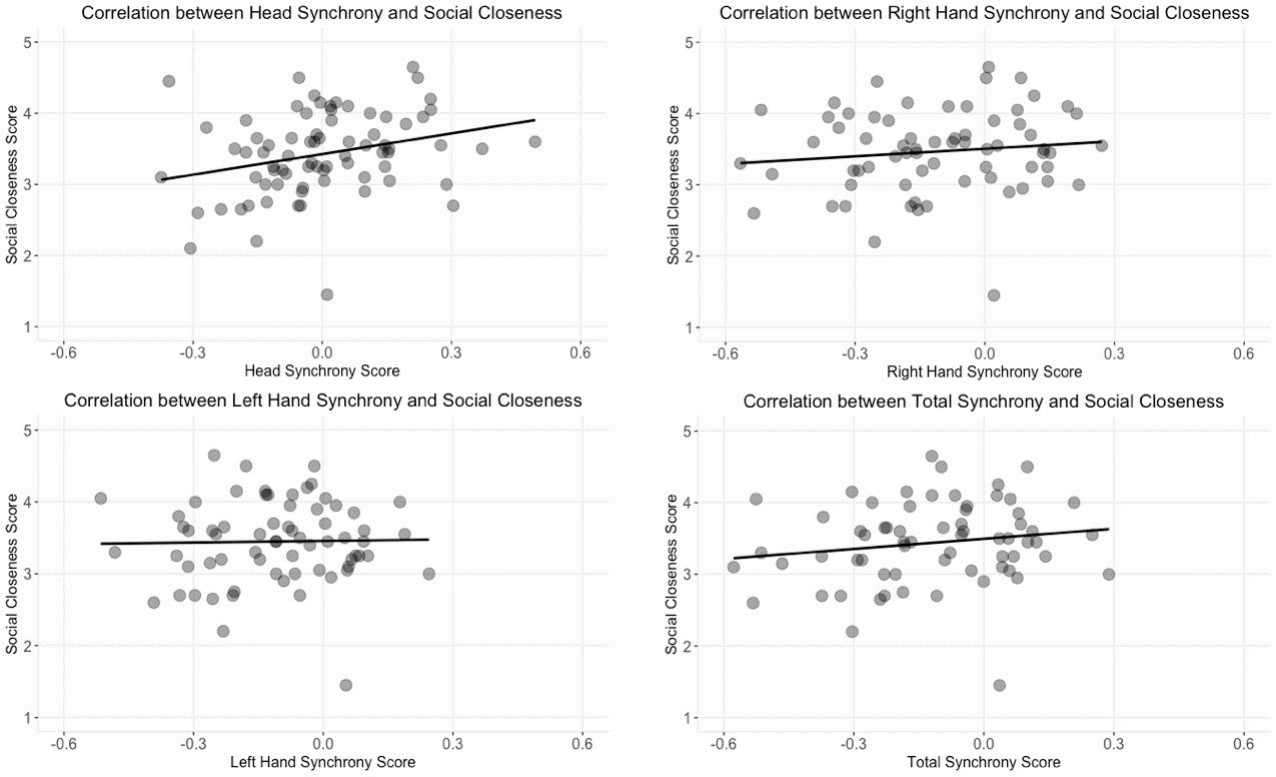
Synchrony and social closeness. This figure shows the correlation between synchrony scores from different body regions and social closeness. The top left plot shows the relationship with head synchrony; top right plot shows the relationship with total synchrony; the bottom left plot shows the relationship with left hand synchrony; the bottom right plot shows the relationship with right hand synchrony.

Previous literature has established a correlation between synchrony and rapport [16]. In this study, we wanted to explore whether there was a significant difference between social closeness across different conditions. We also wanted to examine whether the social closeness score would be correlated with the synchrony score. We ran a Wilcoxon rank sum test and found that there was no significant difference between social closeness scores for the avatar conditions and the cube conditions (*W* = 739.5, *p* = 0.823) or between the collaboration and competition conditions (*W* = 758.5, *p* = 0.708). However, there was a significant relationship across conditions between social closeness and synchrony. Using Spearman’s correlation coefficient, we found a significant positive correlation between head synchrony and social closeness (*S* = 49113, *p* = 0.004, *rho* = 0.329), although not for left hand synchrony (*S* = 48321, *p* = 0.774, *rho* = 0.036) or right hand (*S* = 45568, *p* = 0.290, *rho* = 0.130); or total synchrony (*S* = 37281, *p* = 0.140, *rho* = 0.185). Plots of all four data sets are seen in Fig 18.

#### Synchrony, social presence, and self-presence

We also explored the relationships between synchrony and self- and social presence. There were no differences between conditions on self-presence or social presence. There was also no relationship between social or self- presence and synchrony (all *p*’s greater than .25).

## Discussion

In this study, we measured the natural occurrence of synchronous nonverbal behavior in participant pairs interacting in virtual reality, using the movement tracking capabilities of consumer virtual reality equipment. In a series of pre-planned comparisons, we found significant differences between actual and simulated (pseudosynchronous) pairs, supporting the hypothesis that naturally emergent nonverbal synchrony, or turn-taking behavior can be detected in social interactions in virtual reality. We did not detect differences between the synchrony of the collaborative and competitive pairs. Surprisingly, we also found no effect of avatar appearance. There was no difference in synchrony between the two conditions (realistic avatar and cube) both of which preserved gesture and proximity information but differed in realism. In exploratory data analyses, we found positive correlations between synchrony and self-reported social closeness averaged between participants. However, we did not find significant relationships between synchrony and social presence or self-presence.

We found no differences in synchrony between the collaborative and competitive conditions. We note that while participants were verbally instructed to compete with each other in the competitive condition, they may have viewed this competition in a playful light, since they reported no difference in social closeness between competitive and collaborative conditions. In our measures of “synchrony” we actually saw negative correlations between participant movements, which may indicate turn taking behavior.

In our findings, the movement data from hand controllers showed stronger correlations between participants than the data from the head-mounted display, although these correlations were negative. We speculate that this is due to the inhibiting effects of wearing an HMD, which may change participants’ head movements. This raises the question of whether and how nonverbal behavior may be altered in virtual environments. While we did find evidence of participants’ nonverbal behavior being linked, the negative correlations may be more closely related to turn-taking behavior. Thus, it is possible that the behavior we captured in virtual reality is not identical to synchrony that has been captured in face-to-face interactions; although we note that we still found relationships between movement and social closeness, reflecting previous associations with rapport in face-to-face interactions.

We found no differences in nonverbal synchrony between appearance conditions; implying that participants were not affected by whether they had a human appearance or appeared as a shapeshifting cube. The sense of embodiment could be critical to participant engagement in tasks where they were represented by avatars in the virtual environment. It not only impacts how participants perceive themselves and others in virtual reality, but also influences how they interact with their partners and handle different tasks. However, how much “body” is sufficient for participants to feel embodied remains an important ongoing research topic. In this study, we propose two explanations for this. First, it is possible that the cube provided sufficient information on gesture and posture for participants to coordinate. Other research has suggested that participants are quick to adapt to novel avatars [46]; since participants had the opportunity to learn how cubes reflected movement by seeing themselves move in front of a mirror, perhaps the appearance of nonverbal synchrony merely indicates this adaptiveness. An alternative and perhaps more intriguing explanation is that participants may not have relied on visual information to synchronize their movements. When asked to rank what gave them the most information about their conversational partner’s state of mind, most ranked tone of voice first. Further research should investigate participants’ naturally occurring synchrony without any visual feedback, as it is also possible that participants’ may have their synchronized movements may have been based on the cues that they received through their voice. This might also help to explain why the correlations we found between left and right hand movements in particular were negative correlations, representing turn-taking behavior, instead of the positive correlations found in a previous face-to-face study that used the same task [60].

Though previous literature used the absolute values of the correlations of the movement between two participants to derive synchrony scores [61] [10], this method was not included in our study’s pre-registration. We believe that the negative correlations between two participants’ movement captured in our “synchrony measure”, may represent turn-taking behavior, another critical part of social interactions. However, we appreciate the reviewer’s request for this additional analysis using the absolute values. Therefore, we present these results in Table 3 and Table 4 in Appendix A of the Supplementary Materials.

These results show that the difference between the synchrony and pseudosynchrony scores remains, although these differences are not as large as in our pre-planned comparisons. We believe this also supports the contention that these correlations were actually capturing turn-taking behavior, providing further information to help understand how nonverbal behavior in dyads can become entrained.

In our paper, we chose to sum data in increments of 1 to 4 seconds in order to address the issue of missing time stamps or lost data. Another option would have been to use interpolation techniques [62] to predict the value between two data points. However, we chose not to do this but instead to sum up the data within certain time threshold as this is a more conservative measure to handle a small dataset.

## Limitations

Below we list some limitations in our experiment, including sample size, unique population characteristics, and avatar creation limitations.

First, although we used a power analysis on pilot data to set our sample size, the experiment was likely still underpowered because a substantial set of participants’ movement data were discovered to be unusable due to tracking failure during portions of the experiment. Thus, we suggest that the statistically significant interactions found in research questions 3a and 3b should be replicated in a more highly powered experiment. A power analysis suggests a sample size of approximately 100 participants.

Second, despite our best efforts to emphasize competitiveness through the experiment script, participants remained collegial, making the comparison between collaborative and competitive conditions less realistic. In fact, we found no difference between social closeness between conditions, implying that participants may have treated the competition condition more as a friendly game.

Third, the avatar creation process also had limitations. In particular, the ability to customize avatars of different ethnicities were limited by the nature of consumer software and available accessories, such as hair. In addition, the customized yet not fully animated avatar may have contributed to an “uncanny valley” effect [63] [64]. Eye contact and facial expressions are important parts of nonverbal communication. The custom avatar faces created by the consumer software used in this study were not animated and so participants’ avatars did not demonstrate facial animation or eye contact. Thus, participants may have gained less benefit from the avatars than we expected. For example, in the post-experiment survey, several participants mentioned that “The appearance of avatar is designed not that real. It doesn’t have detailed feature of human.” and “The avatars didn’t give an actual sense of the person being there, perhaps partially because the task did not involve actually moving with the avatar?” Furthermore, some participants critiqued the imperfect movement of the avatars when moving around in the virtual space. “The avatars looked a little off in terms of their movement and relative spatial positions of my body parts in relation to each other.”

Finally, in order to customize the avatar bodies and create the avatars on a large scale to run the experiment, we created male and female generic avatars for the networked environment. We also created three skin tones for the avatars. While we approximately matched body and skin tone for participants, we could not completely capture individual differences for all participants; for example, different body types. In addition, some participants may have noticed that the avatar in the networked environment was slightly different from the avatar they embodied in front of the mirror, disrupting the sense of ownership over the custom avatar body. Future work should further examine the effects of customization in avatars on synchrony.

## Next steps

Embodiment in virtual reality is an important area of applied research, but can also help elucidate how embodiment should be. In one condition in this study, we used generic cubes as participants’ avatars to present subtle nonverbal cues in a social interaction. The volume of the cubes changed depending on participants’ hand movement and the orientation of the cubes moved depending on the direction of where the head turned. We did not see large differences in synchrony between the realistic and cube avatar condition. This leads to two new research questions. First, if participants were influenced by the movements of their conversational partner without needing these movements to be linked to an anatomically realistic depiction of a person, this encourages us to explore the boundaries of embodiment further, by creating more expressive nonverbal cues for the cube condition. For example, the colors of the cube could change based on how much participants synchronized with each other, or based on the users’ tone of voice. Second, as mentioned above, we should explore whether participants coordinate movements to the same extent using voice cues alone.

In two of our research questions we asked whether we would see interactions between avatar appearance, task condition, and liking. Indeed, we see that participants in the competitive cube condition did show a statistically significant negative relationship between social closeness and synchrony, and social presence and synchrony. In other words, the more negative the correlations, the higher the participants’ combined ratings of social presence and social closeness. Again, this may indicate a kind of playful turntaking that may be more prevalent in virtual environments, and could possibly be leveraged to increase creativity and social engagement.

Previous literature suggests abundant other ways to detect and analyze synchrony. However, in this study we used summed movement and a simple correlation from a pair’s movements over time to capture a coarse measure of nonverbal synchrony. This method was inspired by researchers such as Paxton and Dale [34] and Ramseyer and Tschacher [10] who measured global body synchrony. We aim to replicate the current study with the same setup in Paxton and Dale’s study to compare the synchrony results from using the video data and the movement data.

Another next step is to explore specific postures using the movement data automatically detected using headsets and hand trackers. This rich dataset may allow us to use the head and hand movement data to understand how specific postures and gestures in conversations in collaborative virtual environments will influence social interactions and open up more research questions. This data can also potentially reveal how synchrony may evolve over time. For example, are participants’ gestures more synchronous in the beginning of an interaction?

## Conclusions

The goal of this study was to detect nonverbal synchrony in dyadic social interactions in virtual reality, understand the effects of avatar appearance on synchrony, and examine the relationship between creativity and synchrony.

Much research remains to be done on the optimal parameters of avatar embodiment for different social tasks, a research area that will surely evolve as users begin to become familiar with the experience of virtual embodiment and develop their own conventions for social behavior. We hope this study will provide useful information to other researchers in this area.

## Data availability statement

We have packaged our data for sharing on a permanent server through Cornell’s Institute for Social and Economic Research. Because movement data can provide sensitive information about individuals [65], we will require an IRB before releasing movement data. Our movement data has been packaged with our processing code so that researchers can exactly replicate our findings or run their own analyses.

## Supporting information

S1 Appendix Appendix A. Tables for summary statistics and Wilcoxon Rank Sum Test for four-second summed distance using Pearson R correlation, and for absolute values of four-second summed distance using Spearman R correlation.
(PDF)

S2 Appendix Script. Includes the script used in the experiment.
(PDF)

S3 Appendix Histograms. Includes the histograms that show the difference in time.
(PDF)

S4 Appendix Histograms. Includes the histograms that show the distribution of the social closeness, self-presence and social presence scores between the four experimental conditions.
(PDF)
