# Supplementary material for: Nonverbal synchrony in virtual reality
Source: PLoS One. 2019 Sep 16;14(9):e0221803. doi: 10.1371/journal.pone.0221803 (PMC6746391; doi:10.1371/journal.pone.0221803)
Supplement: S1 Appendix — Tables for summary statistics and Wilcoxon Rank Sum Test for four-second summed distance using Pearson R correlation, and for absolute values of four-second summed distance using Spearman R correlation. (PDF) [file pone.0221803.s001.pdf]

Appendix A

Table 1. Summary Statistics and Wilcoxon Rank Sum Test for Four-second Summed Distance in Collaborative Condition Using Pearson R Correlation

Body Region	Synchrony	Pseudosynchrony	Wilcox.test
Head (n = 38)	M = 0.040, SD = 0.151	M = 0.009, SD = 0.138	W = 826, p = 0.284
Left Hand (n = 31)	M = -0.061, SD = 0.184	M = 0.042, SD = 0.140	W = 276, p = 0.004
Right Hand (n = 33)	M = -0.044, SD = 0.198	M = 0.006, SD = 0.111	W = 400, p = 0.065
Total (n = 31)	M = -0.064, SD = 0.187	M = 0.023, SD = 0.130	W = 308, p = 0.015

Table 2. Summary Statistics and Wilcoxon Rank Sum Test for Four-second Summed Distance in Competitive Condition Using Pearson R Correlation

Body Region	Synchrony	Pseudosynchrony	Wilcox.test
Head (n = 38)	M = 0.035, SD = 0.196	M = 0.061, SD = 0.141	W = 632, p = 0.355
Left Hand (n = 36)	M = -0.049, SD = 0.140	M = -0.013, SD = 0.121	W = 506, p = 0.111
Right Hand (n = 35)	M = -0.063, SD = 0.197	M = -0.006, SD = 0.173	W = 530, p = 0.338
Total (n = 34)	M = -0.070, SD = 0.189	M = -0.004, SD = 0.156	W = 478, p = 0.224

Table 3. Summary Statistics and Wilcoxon Rank Sum Test for Absolute Values of Four-second Summed Distance in Collaborative Condition Using Spearman R Correlation

Body Region	Synchrony	Pseudosynchrony	Wilcox.test
Head (n = 38)	M = 0.116, SD = 0.085	M = 0.102, SD = 0.066	W = 774, p = 0.595
Left Hand (n = 31)	M = 0.150, SD = 0.123	M = 0.119, SD = 0.073	W = 515, p = 0.635
Right Hand (n = 33)	M = 0.169, SD = 0.115	M = 0.099, SD = 0.077	W = 745, p = 0.010
Total (n = 31)	M = 0.164, SD = 0.125	M = 0.105, SD = 0.075	W = 610, p = 0.069

Table 4. Summary Statistics and Wilcoxon Rank Sum Test for Absolute Values of Four-second Summed Distance in Competitive Condition Using Spearman R Correlation

Body Region	Synchrony	Pseudosynchrony	Wilcox.test
Head (n = 38)	M = 0.145, SD = 0.123	M = 0.111, SD = 0.090	W = 825.5, p = 0.285
Left Hand (n = 36)	M = 0.156, SD = 0.119	M = 0.105, SD = 0.098	W = 835, p = 0.035
Right Hand (n = 35)	M = 0.208, SD = 0.167	M = 0.141, SD = 0.116	W = 747, p = 0.116
Total (n = 34)	M = 0.196, SD = 0.154	M = 0.128, SD = 0.112	W = 736, p = 0.053
