# Supplementary material for: Nonverbal synchrony in virtual reality
Source: PLoS One. 2019 Sep 16;14(9):e0221803. doi: 10.1371/journal.pone.0221803 (PMC6746391; doi:10.1371/journal.pone.0221803)
Supplement: S2 Appendix — Includes the script used in the experiment. (PDF) [file pone.0221803.s002.pdf]

Idea Generation in VR

SCRIPT

(INTRODUCTION)

[When participant arrives at lab]

–Hi, what's your experiment ID?

[check the experiment log to make sure we have the right participant. ***Make sure they are here for Part 2, i.e. they have already get their pictures taken and their participant ID matches the experiment log. If they are here for part 1 to get their picture taken, direct them to RM487]***

[If that's the correct participant,]

–Welcome to the Virtual Embodiment Lab. Feel free to set your stuff down there. Can you please take a moment to make sure your cell phone is completely off, not on vibrate? Thanks.

[After the participants settle down their stuff, direct them to the ***consent form*** *setup on the computer and read this:]*

– Here is the constant form. Please read it carefully before you start the study. **This study is NOT for people who are: prone to motion sickness, or other similar balance and dizziness conditions, as well as people who are pregnant, have had a recent concussion, seizure disorders, history of fainting or seizures, visual impairment, hearing disabilities or any other condition that makes someone prone to dizziness or disorientation. If you have any of the above symptoms or are pregnant, you are NOT eligible to participate in this study.**

–After you read the form, if you agree to participate in the study, please sign the form.

[If one or both of the participants express concerns about their health situations or have sprained ankles, concussion, etc. give both of the participants credits/cash and let them leave. Make a note on the Experiment Log spreadsheet.]

[If the participant chooses to continue the study and fulfills all requirement,]

----Thank you so much for finishing the consent form. Sorry that I have to read from the script because I want to make sure I give every participant the same information.

<u>(Collaboration & Avatar)</u>

--Today you will be controlling an avatar in a virtual world. And you will complete a task to generate ideas with your conversational partner in virtual reality. After you complete the task, you will complete a brief questionnaire asking you about your experience in the virtual environment. **Your avatar appearance will look like yourself.**

--You are going to complete a **COLLABORATIVE** task that will help us look at mediated interaction. You will **COLLABORATE** with your conversational partner to generate as many ideas as possible. Though you won't see your conversational partner face to face while you are talking, but you will see them in virtual reality.

--You will hear fifteen environmental principles from the earphone related to water use. These fifteen principles state the reasons behind them, and then an example of one kind of change in behavior that would illustrate this principle. Then you will be given **<u>five minutes</u>** to brainstorm more ways to not only reduce water, **<u>but also energy use</u>** with your conversational partner. You do NOT have to memorize these, as they are just prompts to give you some basic ideas, but please listen carefully because **<u>in order to make your ideas count, try to avoid repeating the ideas in these 15 principles.</u>**

--Do you have any questions?

--Before the experiment starts, I would like to introduce you to the VR equipment that you will be using today and help you adjust to the immersive environment. If you had VR experience before, bear with me to go through these instructions and exercises as I would like to make sure all participants get the same instructions.

[Direct participants to the sensors and show them where to stand.]

--Here are two sensors. Please make sure you stand in the center of this area where the sensors can detect you.

--Here are two controllers. You will hold it like this. *[Show participants how to hold the controllers and where the buttons are. Buttons X, Y are on the left controller and Buttons A, B are on the right controller]*

--This is the left trigger that you will be using today. *[Show participants the left trigger]*

--When you hold it, you can do any gestures like in the real world when you are having a conversation. *[Show them that they can do any gestures when holding the controllers]*

--Feel free to try it but make sure you don't press anything now.

[Show participants how to hold hand controllers. Make sure they don't press anything.]

--May I take the controllers from you since I need to show you the headset? I will hand them back to you after you put on the headset.

--Here is the headset. *[Loosen all three stripes first.]*

--You can fit your forehead first and pull the stripe back. You can adjust the stripe on the top and both sides. Glasses are fine. Here is the headset cover. Feel free to put that on the headset and take time to adjust until you feel comfortable. I will also help you.

[Hand participants the headset, VR Cover and show them how to put the cover on and help participants to adjust the headsets, VR covers or they can adjust it themselves]

[Once they are ready,]

--And I am going to hand you the controllers.

[Hand them controllers after the headset is adjusted.]

[Once the participant put on the headset and hold controllers correctly, ***press "play"*** *of the participant ID* ***"_mirror" project in unity]***

–Now you are in the virtual world. **Please stand straight and press your left trigger.**

[Help participants to make sure they press the correct trigger]

–Please look around to find a mirror.

[Help participants to find the mirror. Once participants found the mirror,]

–You will see yourself in the mirror. Feel free to move around and look at your image in the mirror as well as look down to your own hands, arms and legs.

–If you see a blue grid wall, that is the guardian system. Please move around now to make sure you are away from the wall.

[Once participants check out the avatar]

–Just to help you to adjust to the virtual world, we have three small exercise for you. **Please look at your image in the mirror when you do the exercise.**

–In the first exercise, I will call out a metronome from one to six. When you hear one, raise your left arm, when you hear two put down your left arm and raise your right arm. Repeat the same movement for three to six.

–Are you ready?   One—Two—Three—Four—Five—Six

[Instruct the participants and do the first exercise]

–In the second exercise, I will call out a metronome from one to six. When you hear one, raise up your arms in front of your body. When you hear two, stretch your arms to the side, like a T pose. Repeat the same movement for three to six.

–Are you ready?   One—Two—Three—Four—Five—Six

[Instruct the participants and do the second exercise]

–In the third exercise, I will call out a metronome from one to six. When you hear one, take a step further. When your hear two, take a step back. Repeat the same movement for three to six.

–Are you ready?   One—Two—Three—Four—Five—Six

[Instruct the participants and do the third exercise]

–How do you feel?

–We are going to start the experiment very soon. You will hear this **ending sound** to notify you **the end of the five minutes** when you are working on the idea generation task. *[Play the ending sound for the participant.]*

–You can start to generate ideas as soon as you hear **<u>“You may now begin”</u>** from the instructions in the earphone. Do you have any questions?

[If participants have questions, answer the questions, if they say “no”, continue]

–Are you ready to start the experiment now? *[If participants say “yes”]*

–Please close your eyes now since I am going to switch to another scene to connect you with the other participant now. I will let you know when we are ready.

*[**Unplay** and **close** the unity program. Then open the **participant ID “_experiment”** Unity project by clicking Unity from the bottom menu panel-->* 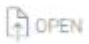

*-->navigate to the directory of where the **participant ID “_experiment”** is saved]*

[Once the Unity interface shows up, ***the server*** ***room 459*** ***has to start to*** ***click play first*** *(wait until you can see things moving in Unity). The RA in RM459 texts RA in RM494 "the server has already started". Then the RA in* ***RM 494 click the "play"*** *button of the* ***participant ID "_experiment"*** *Unity Project.]*

--Now you can open your eyes. **Please stand straight and press your left controller.**

[Make sure the participant pressed the correct trigger]

-- Can you hear each other? Feel free to talk to each other and I will give you some time to adjust to the new environment. Just let me know when you are ready.

[Once participants say they are ready, press the Y button on their left controller for them. The audio instruction will load automatically]

--Can you hear the instruction?

[If yes, then make sure they don't get tripped by the cords. Also watch the Unity window to observe what participants are seeing until 5 minutes end.]

--Thank you so much for finishing this part of the study! We have one more survey for you. *[Direct participants to the survey that has already been set up outside]*

--Are you compensated by cash or credit?

[if cash] feel free to open the door and come back to this room and I will give you cash.

[if credit] feel free to leave after you finish the survey, we will award you credits in 1-2 days.

(Competition & Avatar)

--Today you will be controlling an avatar in a virtual world. And you will complete a task to generate ideas with your conversational partner in virtual reality. After you complete the task, you will complete a brief questionnaire asking you about your experiences in the virtual environment. **Your avatar appearance will look like yourself.**

--You are going to complete a **COMPETITIVE** task that will help us look at mediated interaction. You will **COMPETE** with your conversational partner to generate as many ideas as possible **FOR YOURSELF**. Though you won't see your conversational partner face to face while you are talking, but you will see them in virtual reality.

--You will hear fifteen environmental principles from the earphone related to water use. These fifteen principles state the reasons behind them, and then an example of one kind of change in behavior that would illustrate this principle. Then you will be given **five minutes** to brainstorm more ways to not only reduce water, **but also energy use** with your conversational partner. You do NOT have to memorize these, as they are just prompts to give you some basic ideas, but please listen carefully because **in order to make your ideas count, try to avoid repeating the ideas in these 15 principles.**

--Do you have any questions?

--Before the experiment starts, I would like to introduce you to the VR equipment that you will be using today and help you adjust to the immersive environment. If you had VR experience before, bear with me to go through these instructions and exercises as I would like to make sure all participants get the same instructions.

[Direct participants to the sensors and show them where to stand.]

--Here are two sensors. Please make sure you stand in the center of this area where the sensors can detect you.

--Here are two controllers. You will hold it like this. *[Show participants how to hold the controllers and where the buttons are. Buttons X, Y are on the left controller and Buttons A, B are on the right controller]*

--This is the left trigger that you will be using today. *[Show participants the left trigger]*

--When you hold it, you can do any gestures like in the real world when you are having a conversation. *[Show them that they can do any gestures when holding the controllers]*

--Feel free to try it but make sure you don't press anything now.

[Show participants how to hold hand controllers. Make sure they don't press anything.]

--May I take the controllers from you since I need to show you the headset? I will hand them back to you after you put on the headset.

--Here is the headset. *[Loosen all three stripes first.]*

--You can fit your forehead first and pull the stripe back. You can adjust the stripe on the top and both sides. Glasses are fine. Here is the headset cover. Feel free to put that on the headset and take time to adjust until you feel comfortable. I will also help you.

[Hand participants the headset, VR Cover and show them how to put the cover on and help participants to adjust the headsets, VR covers or they can adjust it themselves]

[Once they are ready,]

--And I am going to hand you the controllers.

[Hand them controllers after the headset is adjusted.]

*[Once the participant put on the headset and hold controllers correctly, **press "play"** of the participant ID **"_mirror" project in unity]***

--Now you are in the virtual world. **Please stand straight and press your left trigger.**

[Help participants to make sure they press the correct trigger]

--Please look around to find a mirror.

[Help participants to find the mirror. Once participants found the mirror,]

--You will see yourself in the mirror. Feel free to move around and look at your image in the mirror as well as look down to your own hands, arms and legs.

--If you see a blue grid wall, that is the guardian system. Please move around now to make sure you are away from the wall.

[Once participants check out the avatar]

--Just to help you to adjust to the virtual world, we have three small exercise for you. **Please look at your image in the mirror when you do the exercise.**

--In the first exercise, I will call out a metronome from one to six. When you hear one, raise your left arm, when you hear two put down your left arm and raise your right arm. Repeat the same movement for three to six.

--Are you ready? One—Two—Three—Four—Five—Six

[Instruct the participants and do the first exercise]

--In the second exercise, I will call out a metronome from one to six. When you hear one, raise up your arms in front of your body. When you hear two, stretch your arms to the side, like a T pose. Repeat the same movement for three to six.

–Are you ready?   One—Two—Three—Four—Five—Six

[Instruct the participants and do the second exercise]

–In the third exercise, I will call out a metronome from one to six. When you hear one, take a step further. When your hear two, take a step back. Repeat the same movement for three to six.

–Are you ready?   One—Two—Three—Four—Five—Six

[Instruct the participants and do the third exercise]

–How do you feel?

–We are going to start the experiment very soon. You will hear this **ending sound** to notify you **the end of the five minutes** when you are working on the idea generation task. *[Play the ending sound for the participant.]*

–You can start to generate ideas as soon as you hear **"You may now begin"** from the instructions in the earphone. Do you have any questions?

[If participants have questions, answer the questions, if they say "no", continue]

–Are you ready to start the experiment now? *[If participants say "yes"]*

–Please close your eyes now since I am going to switch to another scene to connect you with the other participant now. I will let you know when we are ready.

*[**Unplay** and **close** the unity program. Then open the **participant ID "_experiment"***

Unity project by clicking Unity from the bottom menu panel--> 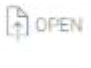 *-->navigate to the directory of where the **participant ID "_experiment"** is saved]*

[Once the Unity interface shows up, ***the server <u>room 459</u> has to start to <u>click play first</u>*** *<u>(wait until you can see things moving in Unity).</u> The RA in RM459 texts RA in RM494 "the server has already started". Then the RA in* ***RM 494 click the "play"*** *button of the* ***participant ID "_experiment"*** *Unity Project.]*

–Now you can open your eyes. **Please stand straight and press your left controller.**

[Make sure the participant pressed the correct trigger]

– Can you hear each other? Feel free to talk to each other and I will give you some time to adjust to the new environment. Just let me know when you are ready.

[Once participants say they are ready, press the Y button on their left controller for them. The audio instruction will load automatically]

–Can you hear the instruction?

[If yes, then make sure they don't get tripped by the cords. Also watch the Unity window to observe what participants are seeing until 5 minutes end.]

–Thank you so much for finishing this part of the study! We have one more survey for you. *[Direct participants to the survey that has already been set up outside]*

–Are you compensated by cash or credit?
[if cash] feel free to open the door and come back to this room and I will give you cash.
[if credit] feel free to leave after you finish the survey, we will award you credits in 1-2 days.

(Collaboration & Cube)

--Today you will be controlling an avatar in a virtual world. And you will complete a task to generate ideas with your conversational partner in virtual reality. After you complete the task, you will complete a brief questionnaire asking you about your experiences in the virtual environment. **Your avatar appearance will look like a cube.**

--You are going to complete a **COLLABORATIVE** task that will help us look at mediated interaction. You will **COLLABORATE** with your conversational partner to generate as many ideas as possible. Though you won't see your conversational partner face to face while you are talking, but you will see them in virtual reality.

--You will hear fifteen environmental principles from the earphone related to water use. These fifteen principles state the reasons behind them, and then an example of one kind of change in behavior that would illustrate this principle. Then you will be given **five minutes** to brainstorm more ways to not only reduce water, **but also energy use** with your conversational partner. You do NOT have to memorize these, as they are just prompts to give you some basic ideas, but please listen carefully because **in order to make your ideas count, try to avoid repeating the ideas in these 15 principles.**

--Do you have any questions?

--Before the experiment starts, I would like to introduce you to the VR equipment that you will be using today and help you adjust to the immersive environment. If you had VR experience before, bear with me to go through these instructions and exercises as I would like to make sure all participants get the same instructions.

[Direct participants to the sensors and show them where to stand.]

--Here are two sensors. Please make sure you stand in the center of this area where the sensors can detect you.

--Here are two controllers. You will hold it like this. *[Show participants how to hold the controllers and where the buttons are. Buttons X, Y are on the left controller and Buttons A, B are on the right controller]*

--This is the left trigger that you will be using today. *[Show participants the left trigger]*

--When you hold it, you can do any gestures like in the real world when you are having a conversation. *[Show them that they can do any gestures when holding the controllers]*

--Feel free to try it but make sure you don't press anything now.

[Show participants how to hold hand controllers. Make sure they don't press anything.]

--May I take the controllers from you since I need to show you the headset? I will hand them back to you after you put on the headset.

--Here is the headset. *[Loosen all three stripes first.]*

--You can fit your forehead first and pull the stripe back. You can adjust the stripe on the top and both sides. Glasses are fine. Here is the headset cover. Feel free to put that on the headset and take time to adjust until you feel comfortable. I will also help you.

[Hand participants the headset, VR Cover and show them how to put the cover on and help participants to adjust the headsets, VR covers or they can adjust it themselves]

[Once they are ready,]

--And I am going to hand you the controllers.

[Hand them controllers after the headset is adjusted.]

*[Once the participant put on the headset and hold controllers correctly, **press "play"** of the participant ID **"_mirror" project in unity]***

--Now you are in the virtual world. Please look around to find a mirror.

[Help participants to find the mirror. Once participants found the mirror,]

--You will see yourself in the mirror. Feel free to move around and look at your image in the mirror as well as look down to your own hands, arms and legs.

--If you see a blue grid wall, that is the guardian system. Please move around now to make sure you are away from the wall.

[Once participants check out the avatar]

--Just to help you to adjust to the virtual world, we have three small exercise for you. **Please look at your image in the mirror when you do the exercise.**

--In the first exercise, I will call out a metronome from one to six. When you hear one, raise your left arm, when you hear two put down your left arm and raise your right arm. Repeat the same movement for three to six.

--Are you ready? One—Two—Three—Four—Five—Six

[Instruct the participants and do the first exercise]

--In the second exercise, I will call out a metronome from one to six. When you hear one, raise up your arms in front of your body. When you hear two, stretch your arms to the side, like a T pose. Repeat the same movement for three to six.

--Are you ready? One—Two—Three—Four—Five—Six

[Instruct the participants and do the second exercise]

--In the third exercise, I will call out a metronome from one to six. When you hear one, take a step further. When your hear two, take a step back. Repeat the same movement for three to six.

--Are you ready? One—Two—Three—Four—Five—Six

[Instruct the participants and do the third exercise]

--How do you feel?

--We are going to start the experiment very soon. You will hear this **ending sound** to notify you **the end of the five minutes** when you are working on the idea generation task. *[Play the ending sound for the participant.]*

--You can start to generate ideas as soon as you hear <u>**"You may now begin"**</u> from the instructions in the earphone. Do you have any questions?

[If participants have questions, answer the questions, if they say "no", continue]

--Are you ready to start the experiment now? *[If participants say "yes"]*

--Please close your eyes now since I am going to switch to another scene to connect you with the other participant now. I will let you know when we are ready.

*[**Unplay** and **close** the unity program. Then open the **participant ID "_experiment"***

Unity project by clicking Unity from the bottom menu panel--> 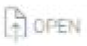

*-->navigate to the directory of where the **participant ID "_experiment"** is saved]*

*[Once the Unity interface shows up, **the server <u>room 459</u> has to start to <u>click play first</u>** <u>(wait until you can see things moving in Unity).</u> The RA in RM459 texts RA in*

RM494 “the server has already started”. Then the RA in ***RM 494 click the “play”*** *button of the* ***participant ID “_experiment”*** *Unity Project.]*

--Now you can open your eyes.

-- Can you hear each other? Feel free to talk to each other and I will give you some time to adjust to the new environment. Just let me know when you are ready.

[Once participants say they are ready, press the Y button on their left controller for them. The audio instruction will load automatically]

--Can you hear the instruction?

[If yes, then make sure they don’t get tripped by the cords. Also watch the Unity window to observe what participants are seeing until 5 minutes end.]

--Thank you so much for finishing this part of the study! We have one more survey for you. *[Direct participants to the survey that has already been set up outside]*

--Are you compensated by cash or credit?
[if cash] feel free to open the door and come back to this room and I will give you cash.
[if credit] feel free to leave after you finish the survey, we will award you credits in 1-2 days.

(Competition & Cube)

--Today you will be controlling an avatar in a virtual world. And you will complete a task to generate ideas with your conversational partner in virtual reality. After you complete the task, you will complete a brief questionnaire asking you about your experiences in the virtual environment. **Your avatar appearance will look like a cube.**

--You are going to complete a **COMPETITIVE** task that will help us look at mediated interaction. You will **COMPETE** with your conversational partner to generate as many ideas as possible **FOR YOURSELF**. Though you won't see your conversational partner face to face while you are talking, but you will see them in virtual reality.

--You will hear fifteen environmental principles from the earphone related to water use. These fifteen principles state the reasons behind them, and then an example of one kind of change in behavior that would illustrate this principle. Then you will be given **five minutes** to brainstorm more ways to not only reduce water, **but also energy use** with your conversational partner. You do NOT have to memorize these, as they are just prompts to give you some basic ideas, but please listen carefully because **in order to make your ideas count, try to avoid repeating the ideas in these 15 principles.**

--Do you have any questions?
--Before the experiment starts, I would like to introduce you to the VR equipment that you will be using today and help you adjust to the immersive environment. If you had VR experience before, bear with me to go through these instructions and exercises as I would like to make sure all participants get the same instructions.

[Direct participants to the sensors and show them where to stand.]
--Here are two sensors. Please make sure you stand in the center of this area where the sensors can detect you.

–Here are two controllers. You will hold it like this. *[Show participants how to hold the controllers and where the buttons are. Buttons X, Y are on the left controller and Buttons A, B are on the right controller]*

–This is the left trigger that you will be using today. *[Show participants the left trigger]*

–When you hold it, you can do any gestures like in the real world when you are having a conversation. *[Show them that they can do any gestures when holding the controllers]*

--Feel free to try it but make sure you don't press anything now.

[Show participants how to hold hand controllers. Make sure they don't press anything.]

–May I take the controllers from you since I need to show you the headset? I will hand them back to you after you put on the headset.

–Here is the headset. *[Loosen all three stripes first.]*

–You can fit your forehead first and pull the stripe back. You can adjust the stripe on the top and both sides. Glasses are fine. Here is the headset cover. Feel free to put that on the headset and take time to adjust until you feel comfortable. I will also help you.

[Hand participants the headset, VR Cover and show them how to put the cover on and help participants to adjust the headsets, VR covers or they can adjust it themselves]

[Once they are ready,]

–And I am going to hand you the controllers.

[Hand them controllers after the headset is adjusted.]

*[Once the participant put on the headset and hold controllers correctly, **press "play"** of the participant ID **"_mirror" project in unity]***

--Now you are in the virtual world. Please look around to find a mirror.

[Help participants to find the mirror. Once participants found the mirror,]

--You will see yourself in the mirror. Feel free to move around and look at your image in the mirror as well as look down to your own hands, arms and legs.

--If you see a blue grid wall, that is the guardian system. Please move around now to make sure you are away from the wall.

[Once participants check out the avatar]

--Just to help you to adjust to the virtual world, we have three small exercise for you. **Please look at your image in the mirror when you do the exercise.**

--In the first exercise, I will call out a metronome from one to six. When you hear one, raise your left arm, when you hear two put down your left arm and raise your right arm. Repeat the same movement for three to six.

--Are you ready? One—Two—Three—Four—Five—Six

[Instruct the participants and do the first exercise]

--In the second exercise, I will call out a metronome from one to six. When you hear one, raise up your arms in front of your body. When you hear two, stretch your arms to the side, like a T pose. Repeat the same movement for three to six.

--Are you ready? One—Two—Three—Four—Five—Six

[Instruct the participants and do the second exercise]

--In the third exercise, I will call out a metronome from one to six. When you hear one, take a step further. When your hear two, take a step back. Repeat the same movement for three to six.

--Are you ready? One—Two—Three—Four—Five—Six

[Instruct the participants and do the third exercise]

--How do you feel?

--We are going to start the experiment very soon. You will hear this **ending sound** to notify you **the end of the five minutes** when you are working on the idea generation task. *[Play the ending sound for the participant.]*

--You can start to generate ideas as soon as you hear **"You may now begin"** from the instructions in the earphone. Do you have any questions?

[If participants have questions, answer the questions, if they say "no", continue]

--Are you ready to start the experiment now? *[If participants say "yes"]*

--Please close your eyes now since I am going to switch to another scene to connect you with the other participant now. I will let you know when we are ready.

*[**Unplay** and **close** the unity program. Then open the **participant ID "_experiment"***

Unity project by clicking Unity from the bottom menu panel--> 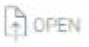

*-->navigate to the directory of where the **participant ID "_experiment"** is saved]*

*[Once the Unity interface shows up, **the server room 459 has to start to click play first** (wait until you can see things moving in Unity). The RA in RM459 texts RA in RM494 "the server has already started". Then the RA in **RM 494 click the "play"** button of the **participant ID "_experiment"** Unity Project.]*

–Now you can open your eyes.

– Can you hear each other? Feel free to talk to each other and I will give you some time to adjust to the new environment. Just let me know when you are ready.

[Once participants say they are ready, press the Y button on their left controller for them. The audio instruction will load automatically]

–Can you hear the instruction?

[If yes, then make sure they don't get tripped by the cords. Also watch the Unity window to observe what participants are seeing until 5 minutes end.]

–Thank you so much for finishing this part of the study! We have one more survey for you. *[Direct participants to the survey that has already been set up outside]*

–Are you compensated by cash or credit?

[if cash] feel free to open the door and come back to this room and I will give you cash.

[if credit] feel free to leave after you finish the survey, we will award you credits in 1-2 days.
