# Supplementary material for: Nonverbal synchrony in virtual reality
Source: PLoS One. 2019 Sep 16;14(9):e0221803. doi: 10.1371/journal.pone.0221803 (PMC6746391; doi:10.1371/journal.pone.0221803)
Supplement: S3 Appendix — Includes the histograms that show the difference in time. (PDF) [file pone.0221803.s003.pdf]

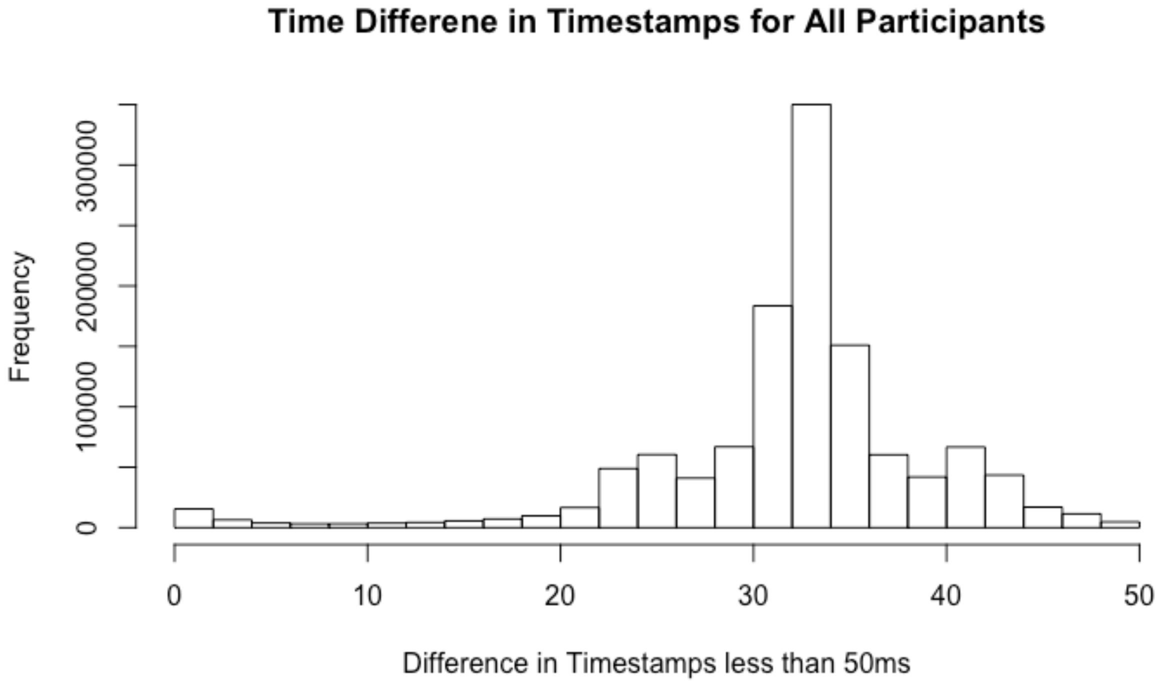
Time Differene in Timestamps for All Participants
Frequency
0
100000
200000
300000
0
10
20
30
40
50
Difference in Timestamps less than 50ms

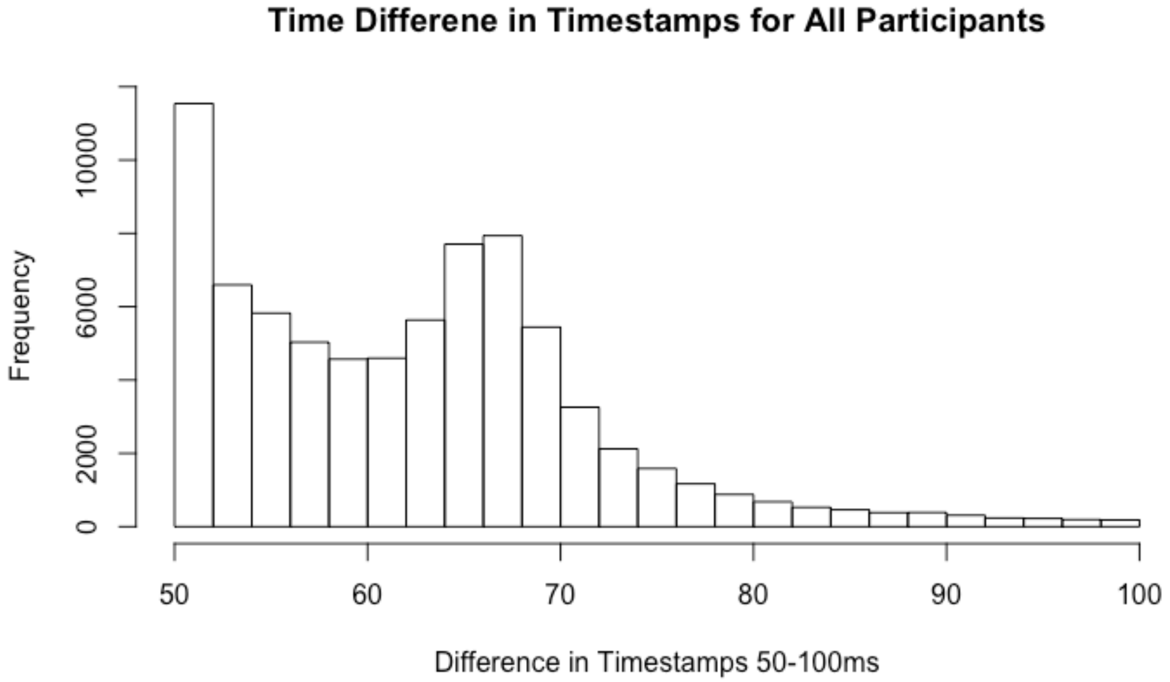
Time Differene in Timestamps for All Participants
Frequency
0
2000
6000
10000
50
60
70
80
90
100
Difference in Timestamps 50-100ms

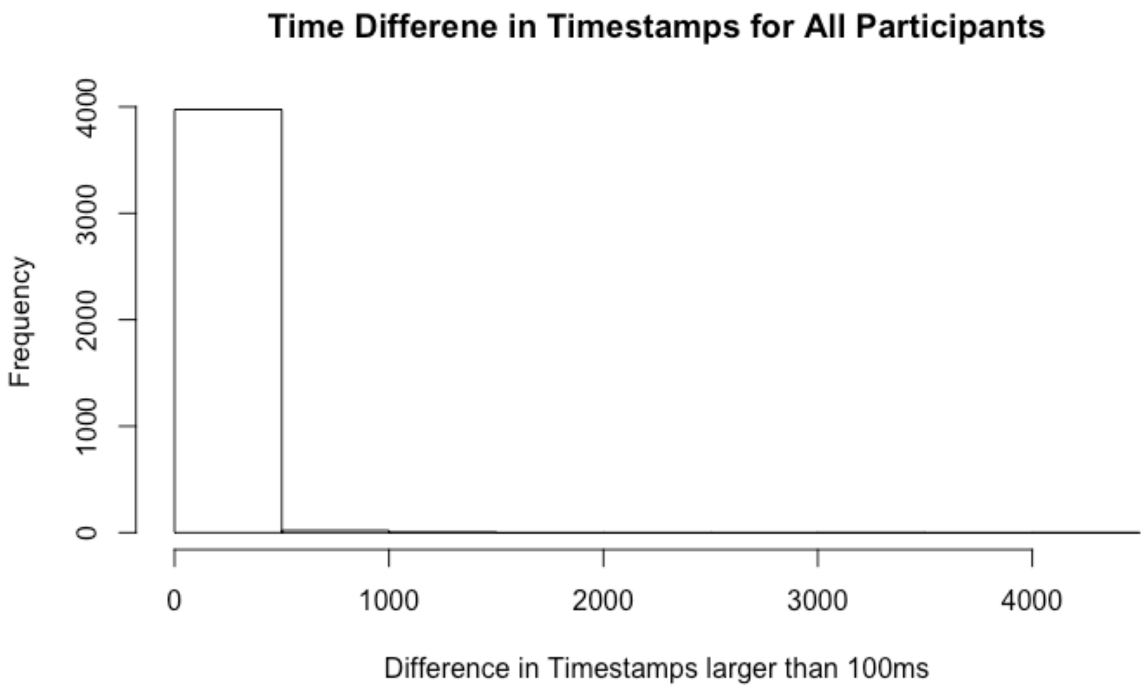
Time Differene in Timestamps for All Participants
Frequency
0
1000
2000
3000
4000
0
1000
2000
3000
4000
Difference in Timestamps larger than 100ms
