# Supplementary material for: Nonverbal synchrony in virtual reality
Source: PLoS One. 2019 Sep 16;14(9):e0221803. doi: 10.1371/journal.pone.0221803 (PMC6746391; doi:10.1371/journal.pone.0221803)

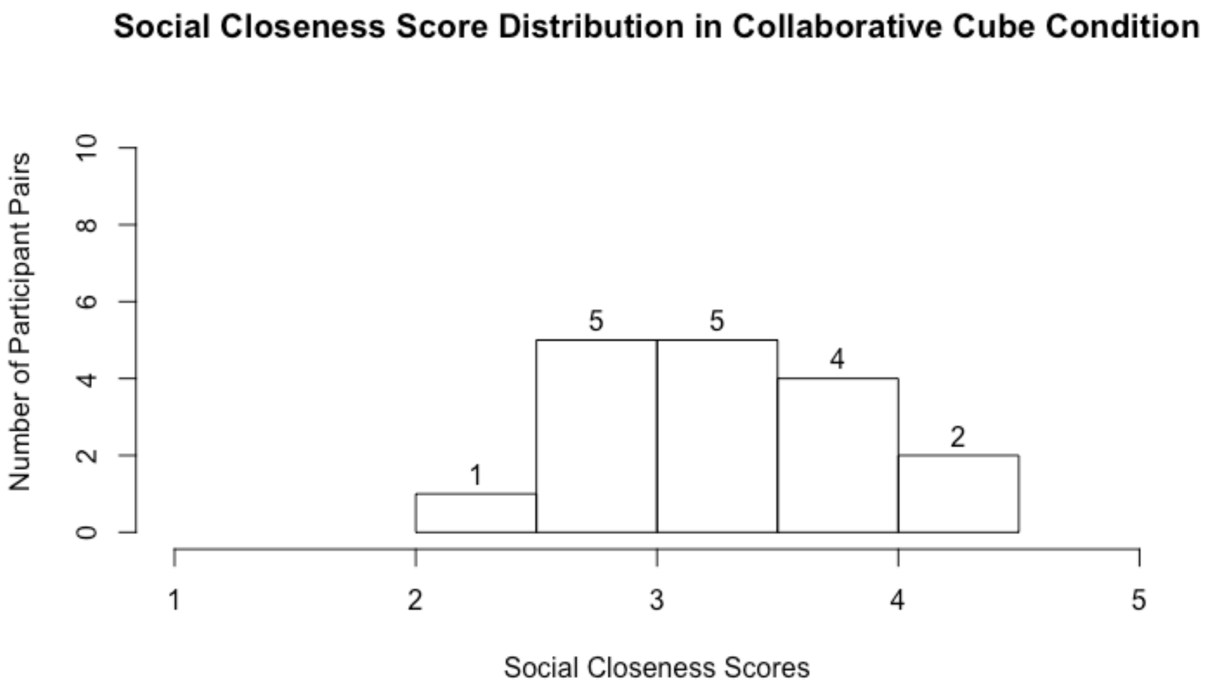

Social Closeness Score Distribution in Collaborative Cube Condition
Number of Participant Pairs
0
2
4
6
8
10
1
5
5
4
2
1
2
3
4
5
Social Closeness Scores

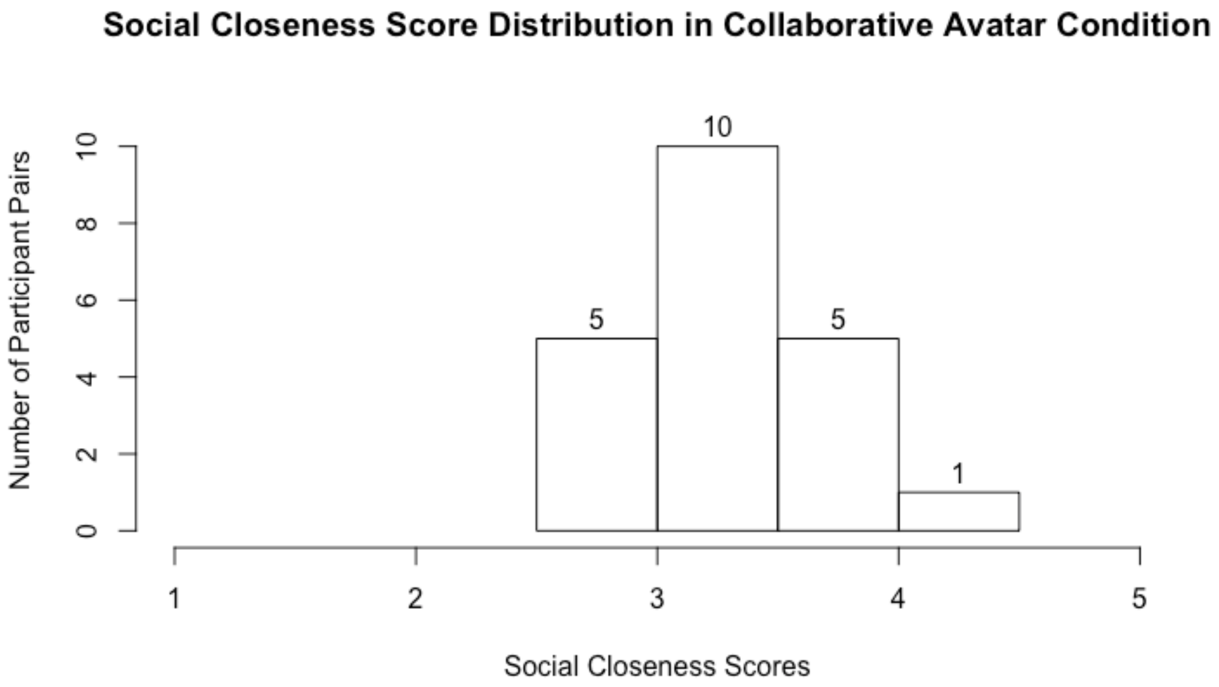

Social Closeness Score Distribution in Collaborative Avatar Condition
Number of Participant Pairs
0
2
4
6
8
10
5
10
5
1
1
2
3
4
5
Social Closeness Scores

Social Closeness Score Distribution in Competitive Cube Condition

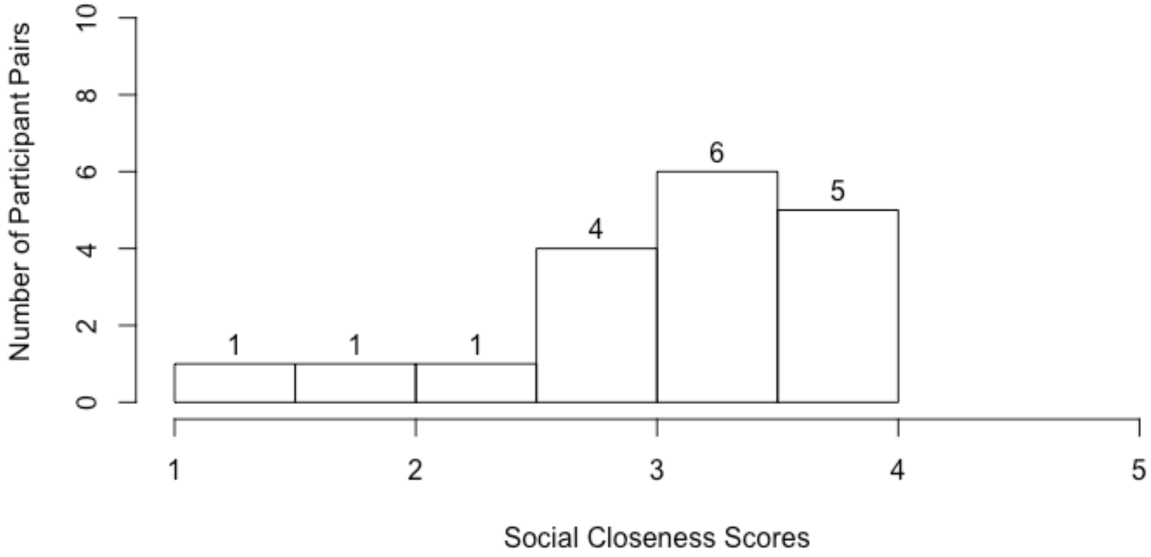

Social Closeness Score Distribution in Competitive Avatar Condition

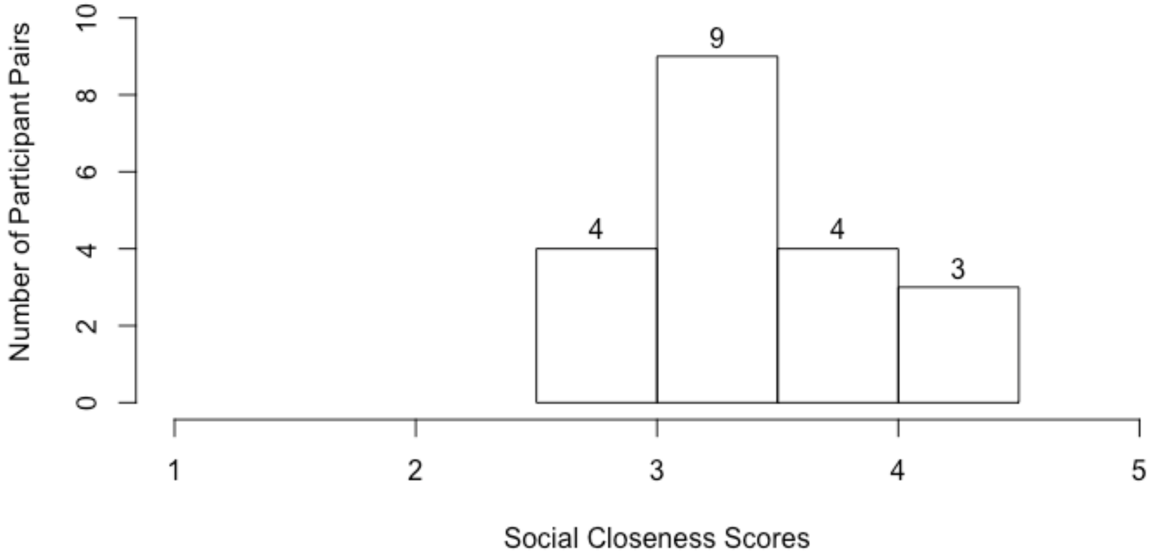

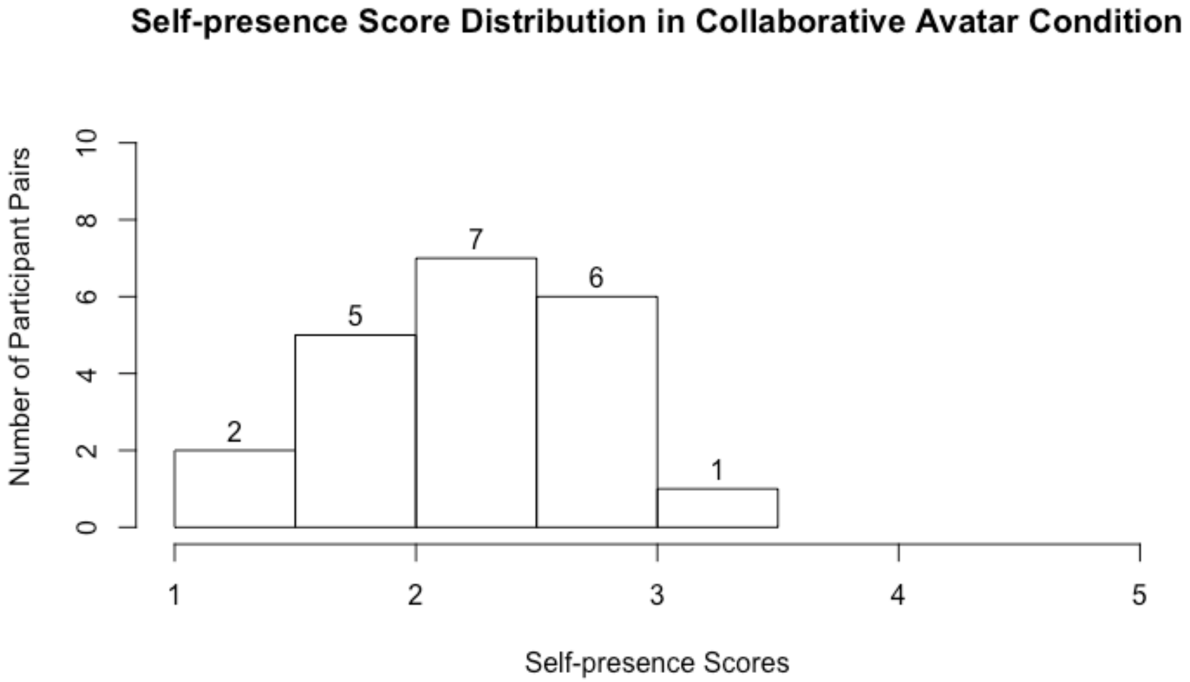
Self-presence Score Distribution in Collaborative Avatar Condition
Number of Participant Pairs
0
2
4
6
8
10
2
5
7
6
1
1
2
3
4
5
Self-presence Scores

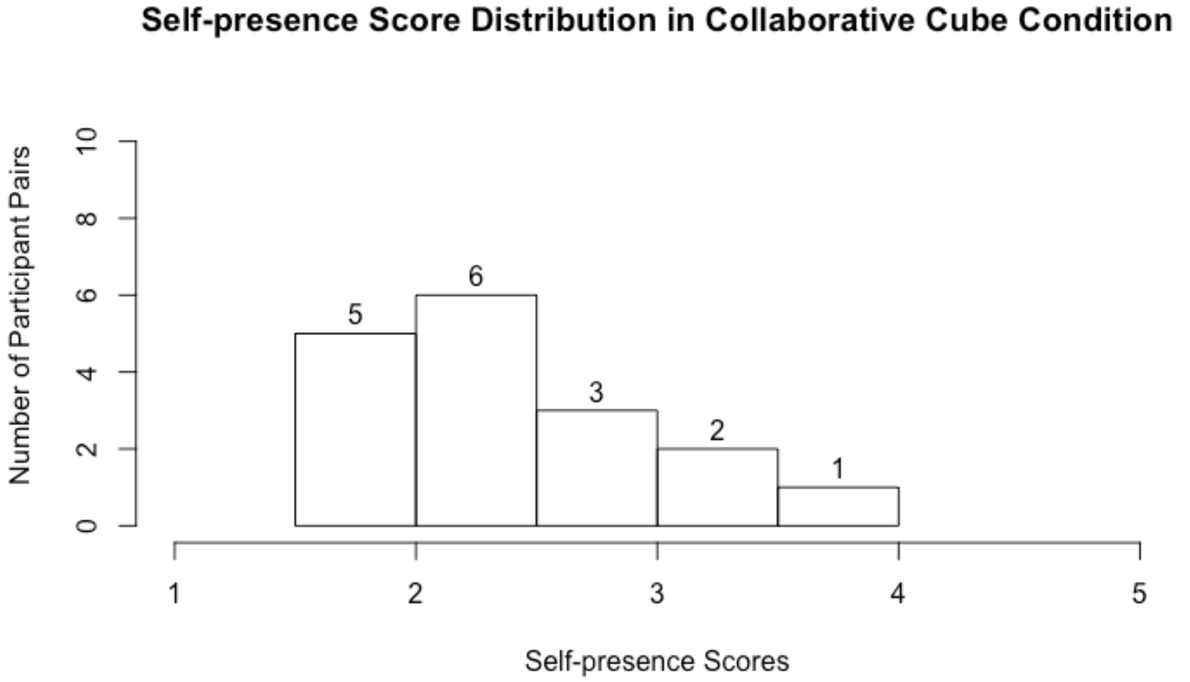
Self-presence Score Distribution in Collaborative Cube Condition
Number of Participant Pairs
0
2
4
6
8
10
5
6
3
2
1
1
2
3
4
5
Self-presence Scores

Self-presence Score Distribution in Competitive Avatar Condition

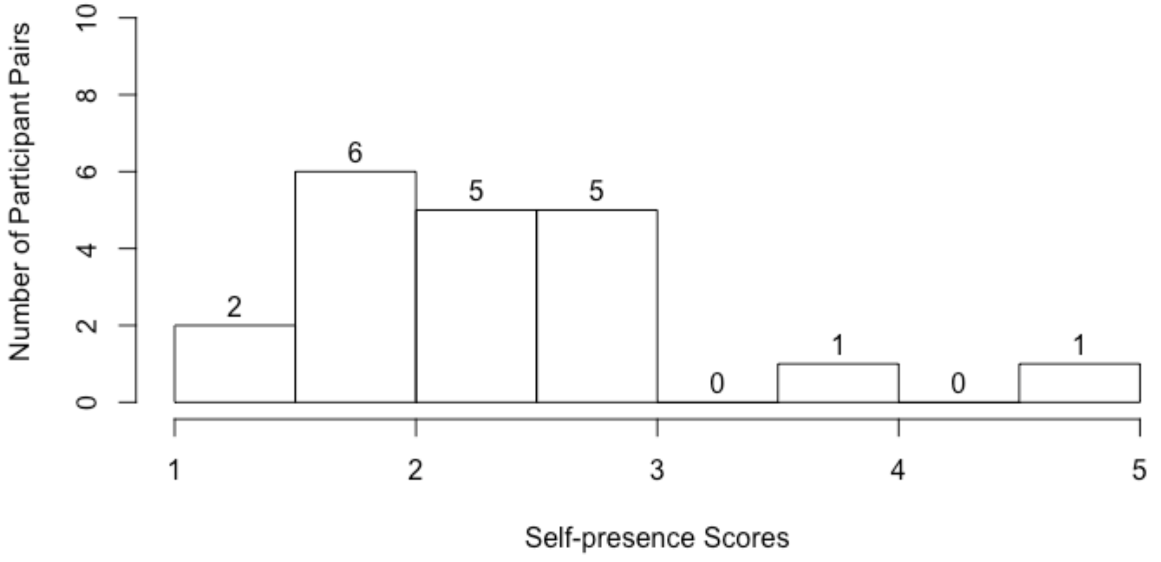

Self-presence Score Distribution in Competitive Cube Condition

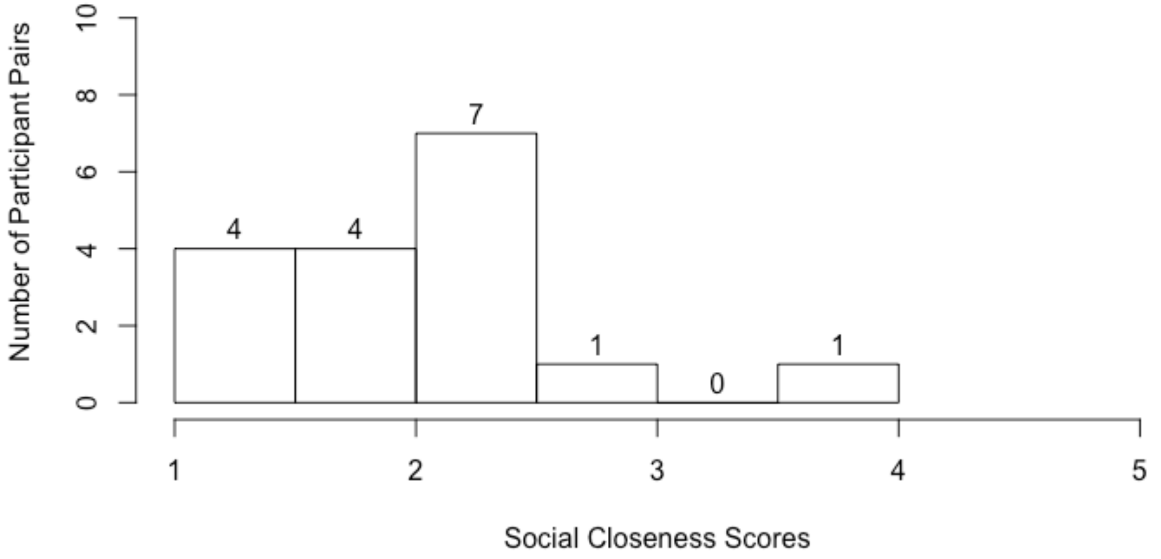

Social Presence Score Distribution in Collaborative Avatar Condition

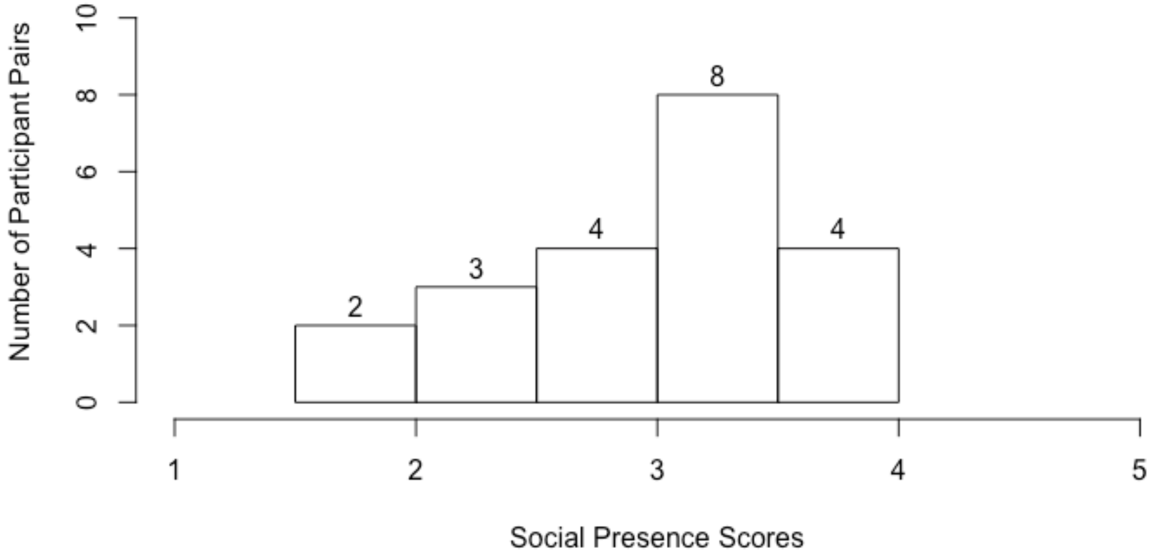

Social Presence Score Distribution in Collaborative Cube Condition

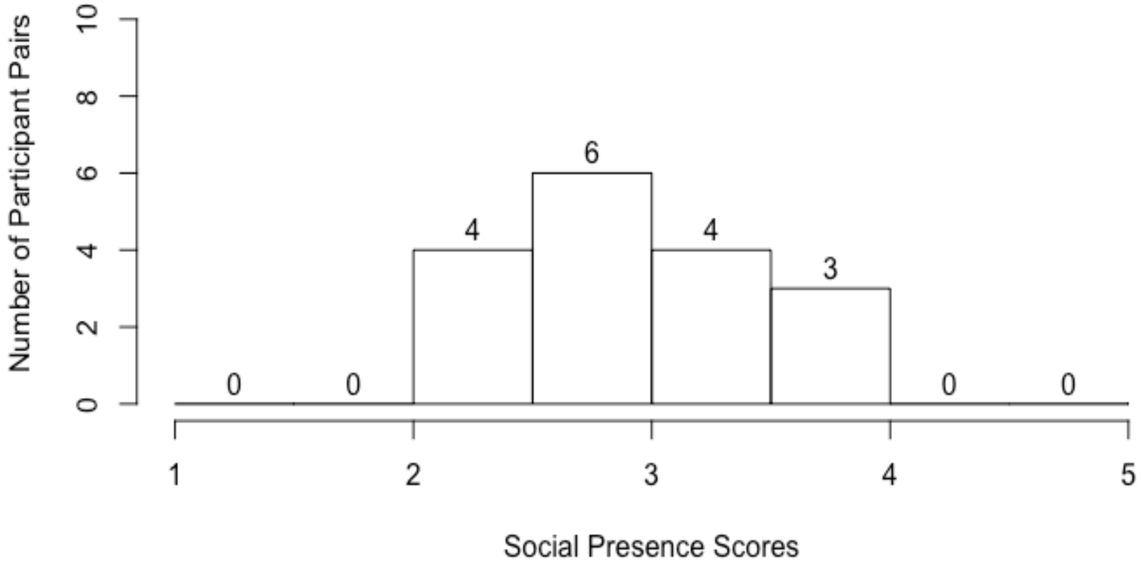

Social Presence Score Distribution in Competitive Cube Condition

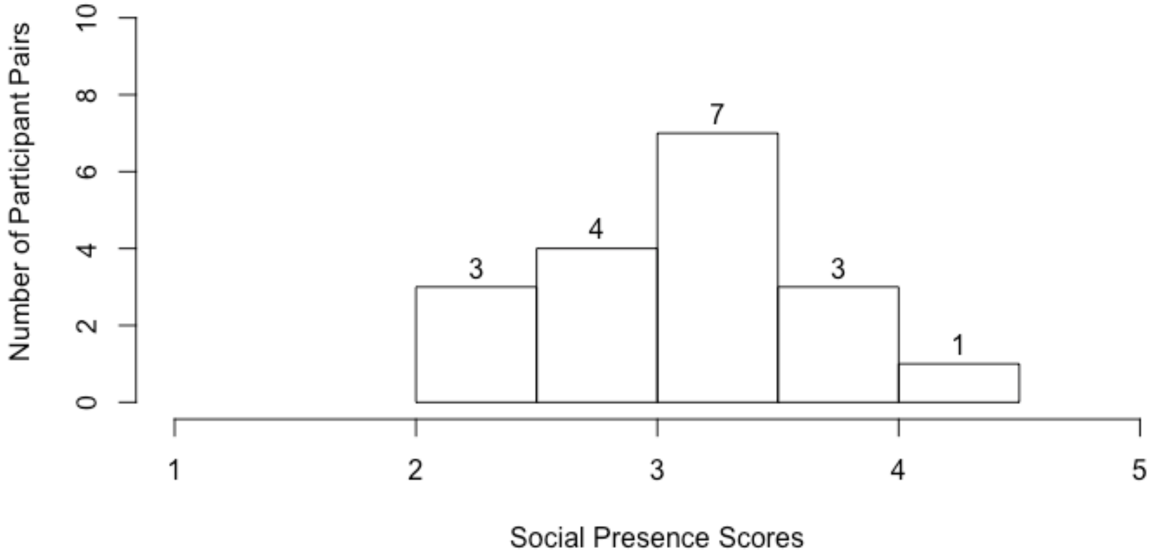

Social Presence Score Distribution in Competitive Avatar Condition

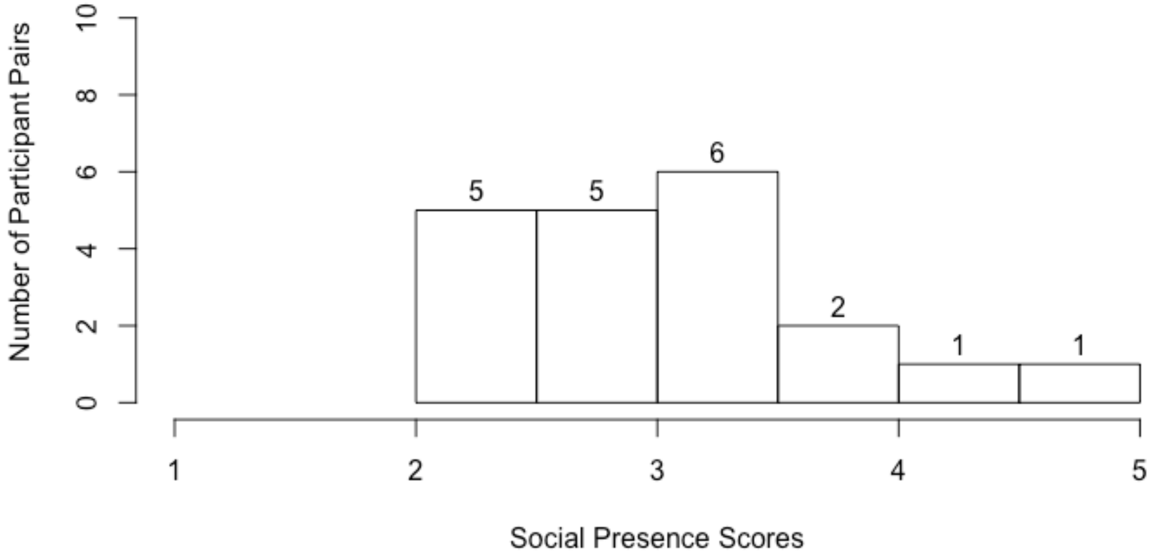

Supplement: S4 Appendix — Includes the histograms that show the distribution of the social closeness, self-presence and social presence scores between the four experimental conditions. (PDF) [file pone.0221803.s004.pdf]
